# Sustainable Synthesis of Food-Grade Emulsifiers from Waste Cooking Oil via Enzymatic Glycerolysis in a Green Solvent System

**DOI:** 10.1021/acs.jafc.5c14323

**Published:** 2026-02-07

**Authors:** Stefano Genualdo, Marina Simona Robescu, Sara Tengattini, Vitiana Cerone, Dhanalakshmi Vadivel, Paola Perugini, Daniele Dondi, Teodora Bavaro

**Affiliations:** † Department of Drug Sciences, 19001 University of Pavia, Viale Taramelli 12, 27100 Pavia, Italy; ‡ Etichub s.r.l., Academic Spin-Off, University of Pavia, Viale Taramelli 12, 27100 Pavia, Italy; § Department of Chemistry, University of Pavia, Viale Taramelli 12, 27100 Pavia, Italy

**Keywords:** monoacylglycerols, enzymatic glycerolysis, lipase transesterification, waste cooking oil, green solvent, food emulsifier, upcycling

## Abstract

The increasing demand for sustainable food-grade emulsifiers has stimulated interest in enzymatic processes utilizing renewable feedstocks. However, the use of waste cooking oil (WCO) for the enzymatic production of monoacylglycerols (MAG) suitable for food applications has been poorly explored. In this study, WCO was successfully upcycled into MAG through lipase-catalyzed glycerolysis using an immobilized enzyme in *tert*-amyl alcohol as a green solvent. Process parameters, including enzyme loading, glycerol-to-WCO molar ratio, temperature, and solvent concentration, were optimized. Under optimized conditions, a MAG yield of 67% was achieved. The resulting MAG exhibited emulsifying properties comparable to those of commercial surfactants in oil-in-water systems. Overall, this work demonstrates a sustainable strategy for the upcycling of WCO into high-value emulsifiers, contributing to circular economy principles in food ingredient production.

## Graphical abstract

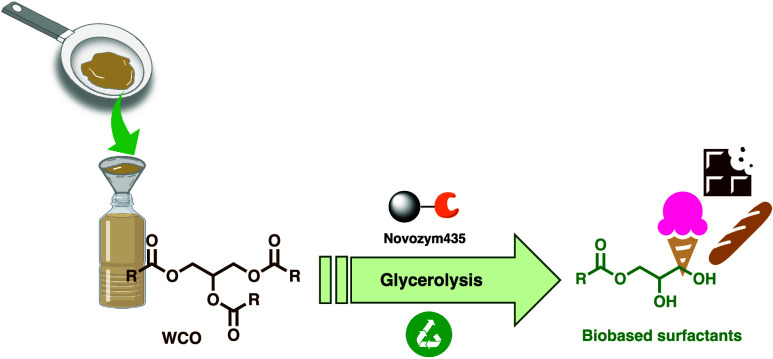

## Introduction

Monoacylglycerols (MAG) are versatile amphiphilic molecules widely used in the food industry as emulsifiers, stabilizers, and texturizers. They play a key role in the formation and stabilization of oil-in-water (O/W) emulsions and contribute to desirable sensory properties such as mouthfeel and consistency.^1–3^ Consequently, MAG are commonly employed in bakery, dairy, confectionery, and margarine formulations due to their ability to enhance product stability and extend shelf life.^1–3^

Industrial production of MAG is typically achieved through chemical glycerolysis of edible oils under high-temperature (220–260 °C) in the presence of alkaline catalysts such as sodium hydroxide or calcium hydroxide.^1–4^ While this approach is well established, it suffers from several drawbacks, including high energy requirements, modest yields (30–50%), and the formation of undesirable byproducts, particularly when unsaturated lipids are processed.^1,3,5^ These issues often require extensive postprocessing, such as molecular distillation, to obtain food-grade MAG quality.^5^ Such limitations highlight the need for more sustainable alternatives to conventional chemical processes.

Enzymatic glycerolysis has emerged as a promising alternative, offering mild reaction conditions, greater regioselectivity, and higher product purity.^1,2,6^ The use of immobilized lipases is particularly attractive because of their reusability and operational stability, aligning well with the principles of green chemistry and sustainable processing.^7^ From a sustainability perspective, solvent-free systems are highly desirable and have been explored in several studies.^2,8–10^ However, in the absence of a reaction medium, the limited miscibility between hydrophilic glycerol and hydrophobic lipid substrates often leads to mass-transfer limitations, resulting in reduced reaction rates and lower MAG yields. Consequently, the efficiency of enzymatic glycerolysis strongly depends on reaction parameters, such as substrate miscibility, enzyme loading, and the choice of reaction medium. In this context, the use of suitable organic solvents or cosolvents has been shown to enhance substrate dispersion and mass transfer, thereby improving overall reaction efficiency.^1,11–14^ Among potential solvents, *tert*-amyl alcohol (TAA) and *tert*-butyl alcohol (TBA) have demonstrated excellent performance in enzymatic systems.^11,15,16^ TAA is classified as a green solvent and it offers several environmental and technical advantages: it is potentially renewable, exhibits low toxicity, and is readily biodegradable.^17^ In addition, its tertiary alcohol structure minimizes esterification side reactions with free fatty acids (FFA) while significantly improving the miscibility between hydrophilic glycerol and hydrophobic oil substrates, thus enhancing mass transfer and reaction efficiency under mild conditions. Although TBA has also been widely explored for similar enzymatic processes due to its favorable polarity and ability to dissolve both reactants, it presents certain operational drawbacks. Specifically, its relatively high melting point (25.7 °C) and narrow liquid range complicate solvent recovery and risk crystallization during process operations. Moreover, its classification as a green solvent is less prominent compared to that of TAA.^11^ For these reasons, TAA was selected in this study as the reaction medium of choice, balancing the process efficiency with environmental compatibility.

In parallel with process sustainability, increasing attention has been devoted to the valorization of waste-derived raw materials. Most reported enzymatic glycerolysis processes use refined, high-purity vegetable oils such as sunflower, palm olein, or marine oils rich in polyunsaturated fatty acids (PUFA).^6^ However, these substrates come with high production costs and significant environmental impacts associated with large-scale agricultural practices. Waste cooking oil (WCO) represents an abundant and low-cost lipid resource whose disposal poses environmental challenges.^5,18^ While WCO has been extensively investigated for biodiesel production, its application in the enzymatic synthesis of food-grade MAG remains limited.^18^ In this context, a clear research gap exists regarding the development of enzymatic processes capable of converting WCO into high-value MAG while preserving functional performance suitable for food applications.

The aim of this work is, therefore, to develop an optimized enzymatic glycerolysis process for the synthesis of MAG from waste catalyzed by an immobilized lipase in TAA as a green solvent. Process parameters were systematically optimized, and the resulting MAG were thoroughly characterized in terms of composition and interfacial properties. By integrating waste valorization, green chemistry principles, and functional performance evaluation, this study proposes an eco-efficient and industrially relevant approach to producing high-performance emulsifiers suitable for modern food systems.

## Materials and Methods

All reactions were performed using WCO collected after use at home or at the restaurant and the lipase Novozym435, kindly provided by Novonesis Lyngby (Lyngby, 2800, Biologiens Vej 2, Denmark). Monoglyceryl oleate (MGO) standard was purchased from Sigma-Aldrich (Milan, MI, Italy). All the other chemicals were purchased from TCI (Cesate, MI, 20031, Via Trebbia 107, Italy) if not otherwise stated. MAG purification was performed by flash chromatography using Silica Gel of high-purity grade, pore size of 60 Å, 70–230 mesh, 63–200 μm (Sigma-Aldrich). Analytical thin layer chromatography (TLC) was performed on silica gel F254-precoated aluminum sheets (0.2 mm layer, Merck, Darmstadt, Germany). Products were detected by spraying with 5% v/v H_2_SO_4_ in ethanol, followed by heating to ca. 150 °C. Lipase activity assay was performed by using a Titrator 718 stat (pH-Stat) Titrino from Metrohm (Herisau, Switzerland). GC–MS analyses were carried out on a Thermo Scientific DSQII single-quadrupole GC–MS system (TraceDSQII mass spectrometer, Trace GC Ultra gas chromatograph, TriPlus Autosampler; Thermo Scientific, San Jose, CA, USA). HPLC analyses were carried out on a VWR Hitachi Chromaster provided by VWR International srl (Milan, MI, 20153, Via San Giusto 85, Italy) and equipped with a 5160 pump, a 5260 auto sampler, and a 5310 column oven. Glyceryl stearate (Geleol) and C10–18 triglycerides solid (Lipocire DM SG) used for the interfacial analysis and formulation process were purchased from Gattefossè (Saint-Priest, 69800, 36 Chem. de Genas, France). Sodium dodecyl sulfate (SDS) used for the interfacial analysis and stearic acid (SA), oleic acid (OA), palmitic acid (PA) used for NIR acquisition were purchased from Sigma-Aldrich (St Louis, USA). Monoolein (>40% purity) was from TCI (Cesate, MI, 20031, Via Trebbia 107, Italy). Glycerol was purchased from VWR International srl (Milan 20153, Via San Giusto 85, Italy). C10–18 triglycerides liquid (Nesatol) and PEG-8 C12–20 alkyl ester (Xalifin 15) were purchased from Vevy Europe (Genoa, GE, 16131, Via Padre Giovanni Semeria, 16A, Italy). Xanthan gum (Keltrol CG), cetyl alcohol, and phenoxyethanol/ethylhexylglycerin (Belguard EHG/PE) were purchased, respectively, from CP Kelco (Atlanta, USA), ACEF (Fiorenzuola d’Arda, PC, 29017, Via Umbria, 8, Italy), and Belchem GmbH (Freiberg, 09599, Ferdinand-Reich-Straße 8, Germany).

### WCO Molecular Weight (MW) Determination

The MW of the WCO mixture was determined using a back-titration method based on the saponification value obtained adapting the ISO 3657 standard method.^19^ WCO was dried with anhydrous Na_2_SO_4_ for 1 day. Afterward WCO was filtered to remove both Na_2_SO_4_ and cooking residues. Filtered WCO (2 g) was reacted with 25 mL of 0.433 M KOH standardized solution in ethanol. The solution was heated to reflux for 20 min to allow the saponification process of the fatty acid chains. After cooling, the unreacted KOH was titrated with a standardized aqueous solution of 0.476 M HCl, using phenolphthalein as an indicator. The MW was then calculated with the formula

$$MW\left(\frac{g}{mol}\right)=3\times \frac{m_{WCO}}{\left({mol}_{KOH}-{mol}_{HCl}\right)}$$

where “factor 3” considers the three fatty acid chains present in each triacylglycerol molecule, each requiring 1 mol of KOH for the saponification. The term mol_KOH_ refers to the total moles of KOH initially added to the reaction mixture, while mol_HCl_ represents the moles of hydrochloric acid used to titrate the unreacted KOH. Therefore, the difference corresponds to the moles of KOH that reacted with the triacylglycerols during the saponification process. The procedure was performed in triplicate.

### Iodine Number Determination

The iodine value was determined by iodometric titration. Approximately 100 mg of oil was weighed into an Erlenmeyer flask and completely dissolved in chloroform. A calibrated pipet was then used to add 5 mL of a 0.2 M ICl solution in acetic acid. The mixture was kept in the dark for 30 min to allow complete reaction with the unsaturated components of the oil. After the reaction period, 10 mL of a 0.5 M KI aqueous solution was added using a calibrated pipet, leading to the quantitative release of iodine from the excess unreacted ICl. The liberated iodine was titrated with a 0.05 M sodium thiosulfate solution until the brown coloration disappeared. At this point, a small amount of starch indicator (iodine indicator, BDH) was added, producing a blue coloration. Titration was continued dropwise with sodium thiosulfate until the solution became completely colorless. A blank determination was performed following the same procedure but without adding the oil sample and without the 30 min reaction time. The volume of sodium thiosulfate consumed in the blank is reported as “blank mL”.

### Oxidation Number Determination

The oxidation number of the oils was determined by the spectrophotometric quantification of triiodide. Briefly, 10 μL of oil was dissolved in 3 mL of a 1:1 (v/v) chloroform–acetic acid mixture. An excess of KI was then added to the solution, and the resulting mixture was diluted 20-fold with the same solvent system. The concentration of I_3_^–^ formed was measured spectrophotometrically at 350 nm. A blank spectrum, prepared using the same amount of oil and solvent but without KI, was recorded and subtracted from the sample spectrum to correct for the background absorbance.

### Determination of Polymerized Triacylglycerols and Total Polar Compounds (TPC)

Polymerized triacylglycerols were quantified by ATR-FTIR spectroscopy following the procedure described in Kuligowski et al.^20^ The TPC content was determined using an ATR-FTIR method according to Chen et al.^21^ Calibration curves were made by using different sunflower oils with known parameters.

### Novozym435 Activity Assay

The activity of Novozym435 was determined using tripropionin as the standard substrate.^15^ The standard reaction mixture was composed of 0.6 mL of acetonitrile, 1 mL of tripropionin, and 18.4 mL of Tris–HCl (25 mM, pH 7.0) and NaCl (100 mM). The reaction was started through the addition of 10–15 mg of Novozym435. The mixture was mechanically stirred, and pH was maintained at 7.0 using standardized 100 mM NaOH as the titrant. Experiments were performed in triplicate. The hydrolytic activity was calculated based on NaOH consumption (mL of NaOH/min). The average activity determined was 1559 U/g ± 257.

### Transesterification of WCO Catalyzed by Novozym435: Preliminary Investigation of Reaction Parameters

Preliminary reactions were setup based on literature data^1,11–14^ to investigate the influence of different parameters on the transesterification outcome. In this preliminary phase different solvents were screened (TBA, TAA, isooctane, glycerol formal, *p*-cymene, 2-methyltetrahydrofuran, methyl *tert*-butyl ether), WCO/glycerol molar ratios (from 1/2 up to 1/10), WCO/solvent weight ratios (from 1/0.75 up to 1/5), and enzyme amounts (31–156 U, 20–100 mg). All of the reactions were incubated at 50 °C (the enzyme optimum working temperature) under orbital shaking (400 rpm) for 24 h. In this preliminary phase, the reactions were monitored by TLC (*n*-hexane/diethyl ether 8:2 with 0.02% formic acid) by spotting 3 μL followed by detection with 5% v/v H_2_SO_4_ in ethanol and heating to ca. 150 °C (Rf_MAG_ = 0; Rf_DAG_ = 0.13–0.2; Rf_FFA_ = 0.35–0.45; Rf_TAG_ = 0.68).

### Design of Experiment (DoE)

A DoE approach was applied to investigate the contribution of various reaction parameters to the production of MAG. A full factorial design (2^*k*^), including all possible combinations of factors and their levels, was selected. Four factors were studied at two levels (resulting in 2^4^ experiments): WCO/solvent ratio (w/w): 1/1 and 1/5; WCO/glycerol ratio (mol/mol): 1/2 and 1/6; enzyme/WCO ratio (w/w, mg/g): 20 and 120; reaction time (h): 6 and 24. The full factorial design scheme is reported in Table 1.

**Table 1:** Full Factorial Design (2^4^ = 16 Experiments) Reporting the Combinations of the Four Studied Parameters, Each Tested at Two Distinct Levels

experiment	WCO/solvent (w/w)	WCO/glycerol (mol/mol)	enzyme/WCO (mg/g)	reaction time (h)
1	1/1	1/2	20	6
2	1/5	1/2	20	6
3	1/1	1/6	20	6
4	1/5	1/6	20	6
5	1/1	1/2	120	6
6	1/5	1/2	120	6
7	1/1	1/6	120	6
8	1/5	1/6	120	6
9	1/1	1/2	20	24
10	1/5	1/2	20	24
11	1/1	1/6	20	24
12	1/5	1/6	20	24
13	1/1	1/2	120	24
14	1/5	1/2	120	24
15	1/1	1/6	120	24
16	1/5	1/6	120	24

Three replicate reactions were also performed under conditions not included in the design matrix (WCO/solvent ratio: 1/3, WCO/glycerol ratio: 1/4, and enzyme/WCO ratio: 70 mg/g) to assess the predictivity of the model.

MGO formation was selected as a representative of the overall yield, and the conversion of MGO (%) in the reaction mixture, calculated with respect to the initial OA content of the oil (45.3%), was considered as a response variable. For this purpose, a calibration curve was built using standard MGO and the HPLC method described in below. The curve, built over the range 0.2 mg/mL–0.7 mg/mL (equation = 4717.4*x* – 646.07, *R* ^2^ = 0.9928, Figure S1), was used to determine MGO concentration, which was then converted to absolute mass by accounting for the reaction volume and subsequently conversion was determined with respect to the initial OA content of the oil (45.3%).

Data analysis was performed using the open-source software Chemometric Agile Tool (CAT), freely available on the site of the Italian Group of Chemometrics (http://www.gruppochemiometria.it).

### Transesterification of WCO Catalyzed by Novozym435: Preparative Scale

WCO (5 g) and glycerol (3.15 g) were suspended in TAA (5 g, 6.17 mL). The mixture was heated for 30 min at 50 °C on an orbital shaker (400 rpm) to prewarm it and have a homogeneous solution. Then the reaction was started through the addition of the enzyme (935 U, 600 mg). After 24 h the enzyme was filtered under vacuum, washed with TAA, and the solvent was distilled under reduced pressure. Subsequently, 4.34 g (*M*_C_) of the resulting crude was purified by flash chromatography (*n*-hexane/ethyl acetate = from 8:2 to 1:1), to obtain the MAG as a white wax (1.76 g, *M*_M_). Since not all of the reaction crude was submitted to flash chromatography, the mass fraction of WCO (*M*_F_) in the crude reaction mixture was first calculated as

$$M_{F}=\frac{m_{WCO}}{m_{WCO}+m_{glycerol}}$$

The gravimetric yield was then determined by comparing the mass of isolated MAG to the theoretical maximum, based on the initial WCO fraction. An isolated yield of 67% was obtained.

$$Yield\left(\%\right)=\frac{M_{M}}{M_{F}\cdot M_{C}}\times 100$$

### Novozym435 Stability in Reaction Conditions

The enzyme stability was evaluated in TAA for 48 h at 50 °C. For each end point a reaction mixture was prepared by incubating Novozym435 (60 mg) in TAA (0.62 mL). After incubation, samples were filtered under vacuum, the enzyme was dried on the filter for 10 min, and its activity was measured by the standard activity assay described before by using ∼20 mg of enzyme derivative. For each end point, the activity has been determined at least in duplicate. The residual activity (%) was calculated as follows

$$R_{A}\left(\%\right)=\frac{Activity\;T_{x}}{Activity\;T_{0}}\times 100$$

### Novozym435 Recycling Study

The enzyme recyclability was performed under the optimal reaction conditions identified by the DoE study. The enzyme was conditioned with TAA and filtered under vacuum. The activity of the conditioned enzyme was determined by the standard activity assay described previously. WCO (10 g) and glycerol (6.3 g) were solubilized in TAA (10 g) and prewarmed at 50 °C and 400 rpm for 30 min. The conditioned enzyme (3.25 g) was added to the reaction mixture and incubated for 24 h at 50 °C. At the end point (24 h) a sample was withdrawn from the reaction mixture and analyzed by HPLC-ELSD to determine MGO conversion. Then, the reaction mixture was filtered under vacuum on a sintered glass filter, and the enzyme was washed with TAA (25 mL) and allowed to dry for 10 min. The enzyme was recovered and used for a subsequent reaction cycle. After some cycles the activity of the catalyst was also checked by the standard activity assay.

### HPLC Analysis

Before HPLC analysis, the samples (2–4 μL; considering the concentration of the reactants in the mixture) were diluted in 1.5 mL of mobile phase A. HPLC analyses were carried out on a Hypersil GOLD C18 column (250 × 4.6 mm, 3 μm) using a linear gradient of eluent A: MeOH/ACN/H_2_O/Formic acid (500:300:198:2) and eluent B: MeOH/Acetone/Formic acid (598:400:2): 0–3 min solvent A 100%; 3–4 min linear gradient to B 100%; 43–60 min re-equilibration with 100% solvent A; flow rate: 1 mL/min. The column temperature was maintained at 60 °C, while the ELSD SEDEX 85LT detector was set at 28 °C, gain 9, and filter 4. The injection volume was 5 μL.

### Monoglyceryl Oleate Purification and Calibration Curve

TLC analysis of the monoglyceryl oleate standard revealed the presence of diglycerides and FFA; therefore, a purification step was performed to ensure a high purity of the analyte in order to use it for the calibration curve. After purification by column chromatography (*n*-hexane/ethyl acetate from 8:2 to 1:1), both monoglyceryl oleate (MGO) and diglyceryl oleate (DGO) were isolated and collected separately. The purity of each fraction was assessed by HPLC analysis (Figure S2). A calibration curve was constructed using the purified MGO within the concentration range of 0.2 mg/mL–0.7 mg/mL (Figure S1).

### Fatty Acid Composition Determination in WCO

In order to determine the FA composition of WCO, FAME were prepared by base-catalyzed transmethylation according to the protocol FIL-IDF 182:1999.^15^ Briefly, a sample of WCO (50 mg), containing the internal standard C19Me (1.25 mL of standard stock solution 10 mg/mL in *n*-heptane), was dissolved in 2.5 mL of *n*-heptane and submitted to direct transesterification with 0.1 mL of KOH/MeOH (10%, w/v) at 40 °C. The sample was vigorously mixed for 1 min, left setting for 5 min, and neutralized by the addition of KHSO_4_ (250 mg). The mixture was centrifuged (3000 rpm for 5 min, centrifuge 5804-R Eppendorf Srl, Milan, Italy) and the supernatant containing the FAME was diluted 1:10 with *n*-heptane and used for gas chromatography–mass spectrometry (GC–MS) analysis.

### Fatty Acid Composition Determination in MAG Samples

In order to determine the FA composition of MAG, FAME were prepared by acid-catalyzed transmethylation according to literature.^15^ MAG (10 mg) were methylated by using 3 mL of H_2_SO_4_/MeOH (10%, v/v) for 1 h at 70 °C. After the addition of 2 mL of NaCl (10%, w/v) and 2 mL of *n*-heptane, and mixing for 1 min, the organic phase was dried with anhydrous Na_2_SO_4_ and centrifuged at 3000 rpm for 5 min. Then the supernatant containing the FAME was diluted 1:10 with *n*-heptane and used for gas chromatography–mass spectrometry (GC–MS) analysis.

### FAME Analysis

Chromatographic analysis of FAME was performed on an Rxi-5Sil MS capillary column (30 m length × 0.25 mm ID × 0.25 μm film thickness; Restek, Milan, Italy) with helium (>99.99%) as the carrier gas at a constant flow rate of 1.0 mL/min. An injection volume of 1 μL was employed. The injector temperature was set at 250 °C, and it was operated in split mode, with a split flow of 10 mL/min. The oven temperature was programmed to range from 45 °C (isothermal for 4 min) to 175 °C (isothermal for 27 min) at a rate of 13 °C/min and then to 215 °C (isothermal for 35 min) at a rate of 4 °C/min. The mass transfer line temperature was set at 250 °C. The total GC running time was 85 min. All mass spectra were acquired with an electron ionization system (EI, Electron Impact mode) with an ionization energy of 70 eV and source temperature of 250 °C. Spectral acquisition was performed in Full Scan mode over a mass range of 35–650 Da. The chromatogram acquisition, detection of mass spectral peaks, and their waveform processing were performed using Xcalibur MS Software, version 2.1 (Thermo Scientific Inc., Waltham, MA, USA). The assignment of chemical structures to chromatographic peaks was based on a comparison with the databases for the GC–MS NIST Mass Spectral Library (NIST 08) and Wiley Registry of Mass Spectral Data (eighth Edition). The percentage content of each component was directly computed from the peak areas in the GC–MS chromatogram.

### NIR Analysis

Both purified and nonpurified MAG were analyzed using NIR spectroscopy for a further nondestructive characterization. Commercial Geleol, monoolein (>40% purity), SA, OA, PA, glycerol were also tested. All samples were dissolved in semisolid vaseline (50 wt %). A blank of vaseline was also prepared. The samples were subjected to NIR acquisition in a range between 950–1650 nm (MicroNIR, VIAVI Solution Inc., USA) using diffuse reflectance mode with an integration time of 0.1 ms and 100 scan count. In the NIR region, each constituent of the complex organic mixture has unique absorption properties, due to the stretching and bending vibrations in molecular bonds.^22,23^ The spectra were subjected to a data pretreatment with Unscramble software ver 10.3.1. Principal Component Analysis (PCA) was performed on the obtained spectra in order to determine the ability of the NIR measurement system to discriminate different samples. The spectra were pretreated by using a standard normal variation followed by the first derivative with Savitzky-Golay smoothing.

### Interfacial Tension Measurements

The interfacial activity of MAG was evaluated through two complementary techniques: direct measurement of O/W interfacial tension, through the Du Noüy ring method, and monitoring of the height variations of the O/W interface by Multiple Light Scattering Analysis.

Both purified and nonpurified MAG were dissolved in hot sunflower oil and brought into contact with water in a 1:1 v/v ratio. Interfacial tension measurements were performed using a tensiometer (K6, Krüss Scientific), with commercial Geleol used as a reference standard. MAG concentrations in the oil phase were adjusted to 0.05 and 0.1 wt %. The interfacial tension values were recorded at 25 °C and expressed in dyn/cm.^24,25^

To further assess interfacial stability, the variation in oil/water interface height was measured using Multiple Light Scattering Analysis (Turbiscan MA 100, Formulaction SA, L’Union, France). The technique employs a near-infrared light source (880 nm) that penetrates the sample. Photons are scattered multiple times by suspended particles or droplets and then detected by two sensors positioned at 0° (transmission, for transparent samples) and 135° (backscattering, for opaque samples) relative to the light source. The optical reading head scans the full height of the sample, collecting transmission and backscattering data every 40 μm, enabling detection of various physical instability phenomena (e.g., creaming, sedimentation, coalescence). The resulting curves report light intensity (as a percentage relative to reference) as a function of sample height (in mm).

Samples for Turbiscan analysis were prepared by mixing 20 g of the O/W formulation in sealed glass vials, at MAG concentrations of 0.05, 0.1, and 1.0 wt %. Both purified and nonpurified MAG were tested. Commercial Geleol and SDS were included as reference surfactants. Measurements were conducted at 40 °C for 2 h, with data acquisition every 15 min.

### Formulation and Stability Evaluation

In order to evaluate the emulsifying properties of MAG, O/W emulsions were prepared with the ingredients listed in Table 2. The surfactant component was either commercial Geleol (used as a reference standard), the purified MAG sample, or the nonpurified MAG mixture.

**Table 2:** Emulsion Composition

phase	ingredients	%
lipophilic phase	cetyl alcohol	2
	C10–18 triglycerides	23
surfactants	surfactant	2
	PEG-8 C12–20 Alkyl Ester	3
hydrophilic phase	xanthan gum	0.2
	phenoxyethanol/Ethylhexylglycerin	1
	water	until 100

First, the hydrophilic phase was prepared by dispersion of xanthan gum in hot water. Subsequently, the lipophilic phase was prepared with the two surfactants (PEG-8 C12–20 Alkyl Ester and the desired surfactant to be studied: Geleol standard, purified MAG or nonpurified MAG, respectively). At this point, the lipophilic and the hydrophilic phases were separately heated at 70 °C. The oil phase was slowly added to the water phase and mixed with a homogenizer to room temperature, after which phenoxyethanol/ethylhexylglycerin was added as a preservative. To assess their stability, the emulsions were analyzed by multiple light scattering (Turbiscan Tower, Formulaction Inc., USA) over a period of 3 days at 40 °C, with measurements taken every 30 min^26,27^

## Results and Discussion

### WCO Characterization

The lipid profile of WCO used in this study was first analyzed by thin-layer chromatography (TLC). The results showed that WCO is mainly composed of triacylglycerols (TAG), with minor amounts of FFA, monoacylglycerols (MAG), and diacylglycerols (DAG) (Figure S3). The HPLC profile of WCO (1 mg/mL) showed the presence of five different species of TAG (Figure S4). The fatty acid composition was determined by gas chromatography coupled with mass spectrometry (GC–MS) in collaboration with Centro Grandi Strumenti of the University of Pavia. Prior to analysis, WCO sample was subjected to a transmethylation reaction using KOH in methanol.^15^ As shown in Figure 1, WCO exhibits a high content of unsaturated fatty acids (81%), mainly consisting of OA (C18:1*n*-9; 45.3%) and linoleic acid (LA, C18:2*n*-6; 35.7%). Saturated fatty acids account for approximately 14.8% of the total, primarily PA (C16:0; 9.5%) and SA (C18:0; 5.3%). Minor quantities of other long-chain unsaturated fatty acids were also detected (4.2%).

**Figure 1:**
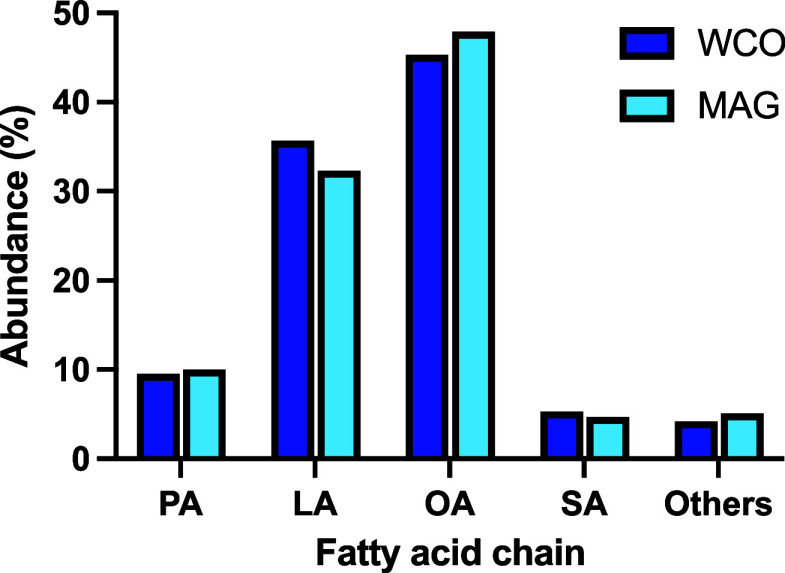
Fatty acid composition of WCO (blue) and MAG (cyan).

The average MW of WCO was determined using a back-titration method based on a base-catalyzed saponification reaction. The resulting value was 877.5 g/mol ± 5.5, which is consistent with the predominance of triacylglycerols as the main lipid species in WCO. The iodine number of the WCO used was determined by the Wijs method to be 120 ± 5. The peroxide number of the oil, determined spectrophotometrically was 70 ± 5 m_eq_ O_2_. Saponification values were around 200 ± 10 mg of KOH/kg of oil. The FTIR analysis showed that the amount of polymerized triacylglycerols in the samples was below 3%. The ATR-FTIR determination of TPC yielded a TPC value of 15 ± 2%. ATR-FTIR spectrum of WCO used, compared to reference sunflower oils is shown in Figure S5. The first derivatives of the spectrum used for data analysis are shown in Figure S6.

### Preliminary Screening of Transesterification Reaction Conditions

Starting from literature,^1,6,11–14^ an initial reaction was performed using WCO (1 g), TBA as solvent, with a WCO/solvent weight ratio of 1/5, a WCO/glycerol molar ratio of 1/5. Novozym435 (200 mg, 312 U) was added as biocatalyst. The reaction mixture was incubated at 50 °C under orbital shaking (400 rpm) for 24 h. TLC monitoring (*n*-hexane/diethyl ether 8:2 with 0.02% formic acid) revealed the formation of a significant amount of FFA, probably due to excessive enzymatic hydrolysis (Figure S7A), suggesting that the amount of enzyme should be reduced. To address this, another reaction was carried out using WCO (0.5 g), while maintaining all other experimental parameters unchanged, except for a 5-fold reduction in the enzyme amount (20 mg, 31 U). Under these conditions the transesterification reaction was favored over hydrolysis as evidenced by the reduced formation of FFA (Figure S7B). Subsequently, the amount of enzyme was kept constant at 20 mg, and a more extensive screening of solvents was performed. Among the tested solvents, TAA provided the best results, enabling efficient conversion of TAG into the corresponding DAG and MAG. TBA and methyl *tert*-butyl ether showed also a decrease in TAG content and formation of mostly DAG and small amounts of MAG. While for isooctane, *p*-cymene and 2-methyl THF some products could be detected but very low consumption of TAG was observed (Figure S8). Once TAA was chosen as the best solvent, different WCO/glycerol molar ratios (from 1/2 to 1/10) were evaluated. As shown in Figure S9, increasing the glycerol content led to enhanced formation of MAG and DAG. In all tested molar ratios, almost complete consumption of TAG was observed after incubation for 24 h. Finally, the impact of solvent quantity was investigated by reducing the WCO/solvent weight ratio from the initial 1/5 to 1/2.5 (50% reduction), 1/1.25 (75%), and 1/0.75 (85%). As can be observed in Figure S10, after 24 h of incubation, the reaction with 50% solvent reduction showed comparable product formation to the standard conditions. However, the reactions where the solvent was reduced by 75% and 85% resulted in lower product yields, despite the near-complete consumption of TAG in all cases.

This preliminary screening allowed us to select the best solvent for the transesterification reaction, TAA, and to understand the influence of several parameters (i.e., amount of enzyme, WCO/glycerol ratio, and WCO/solvent ratio) on the reaction outcome. Based on these results, we were able to define the minimum and maximum ranges of the most relevant parameters to be explored in the DoE study.

### Optimization of Reaction Conditions by DoE

A DoE approach was applied to investigate the contribution of four different reaction parameters to the production of MAG, namely: the WCO/solvent ratio (*x*_1_), the WCO/glycerol ratio (*x*_2_), the enzyme/WCO ratio (*x*_3_), and the reaction time (*x*_4_). A full factorial design (2^*k*^), including all possible combinations of factors and their levels, was selected, resulting in 16 experiments (the full factorial design scheme is reported in Table 1). The conversion of WCO into MGO was considered as the response variable and was quantified by HPLC-ELSD analysis of the reaction mixtures. A summary of the tested conditions and resulting responses is reported in Table S1.

The analysis of variance (ANOVA) and the Pareto chart revealed that all main effects significantly influence the reaction outcome (*p* < 0.05), whereas two-factor interaction terms were found to be statistically nonsignificant (*p* > 0.05) and detrimental to the model’s predictive capability (*Q* ^2^, see Table S2 and Figure S11). Therefore, a reduced linear model including only significant main effects was adopted.

The bar chart of model coefficients for the four main variables, showing coefficient values, their signs, and confidence intervals (** = *p* ≤ 0.01; *** = *p* ≤ 0.001), together with the equation of the reduced model, is reported in Figure 2A. The ANOVA results for the refined model are summarized in Table S3, and the model reliability is further supported by the good correlation between experimental and predicted yields, as illustrated in Figure S12.

**Figure 2:**
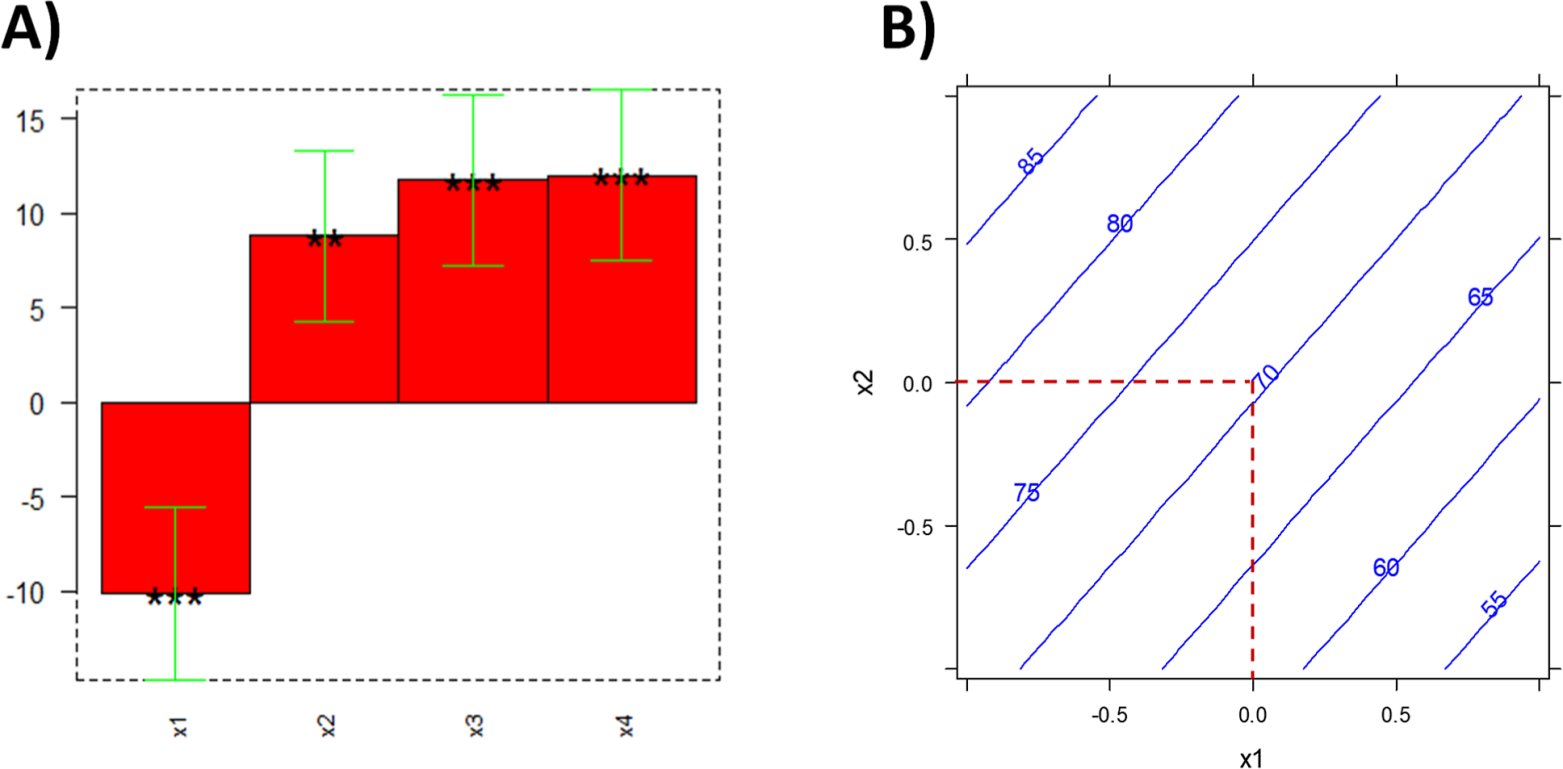
(A) Coefficients and significance obtained from the reduced model based on the full factorial design. The height of each bar (*y*-axis) represents the value of the corresponding coefficient. Whiskers indicate the confidence interval calculated for each coefficient, while asterisks denote statistical significance: **p ≤ 0.01, ***p ≤ 0.001. Model equation: y = 58.61–10.10*x*_1_ + 8.84*x*_2_ + 11.76*x*_3_ + 12.01*x*_4_; (B) 2D contour plot showing the effect of WCO/solvent ratio (*x*_1_) and WCO/glycerol ratio (*x*_2_) on percentage conversion, with enzyme/WCO ratio (*x*_3_) set at 0 (70 mg/g) and reaction time (*x*_4_) set at 1 (24 h). The intersection of the two dotted lines represents the central point of this experimental domain.

The WCO/solvent ratio (*x*_1_) has a negative effect on MGO formation, suggesting that reducing the amount of solvent favors monoglyceride synthesis. This observation may be rationalized by the fact that higher concentrations of solvent can dilute the reactants, thereby reducing their effective molarity and limiting enzyme–substrate interactions. Thus, the improved performance observed at lower solvent levels may reflect a more favorable microenvironment for the biocatalyst and a higher local concentration of substrates.

Conversely, the other three parameters showed positive effects, with the enzyme/WCO ratio (*x*_3_) and reaction time (*x*_4_) having the greatest and most comparable levels of significance.

The moderate yet positive effect of the WCO/glycerol ratio (*x*_2_) indicates that a higher amount of glycerol may enhance the reaction, possibly due to improved substrate availability and better dispersion of glycerol in the reaction medium, which may facilitate enzyme–substrate interactions and shift the equilibrium toward monoglyceride formation.

As expected, a higher enzyme load (*x*_3_) enhances the reaction. Similarly, the positive influence of reaction time (*x*_4_) suggests that the reaction is not complete at 6 h and significantly progresses between 6 and 24 h. Figure 2B shows the 2D contour plot for the WCO/solvent ratio (*x*_1_) and WCO/glycerol ratio (*x*_2_), with the enzyme/WCO ratio fixed at the midpoint between its low and high levels (70 mg/g) and reaction time set to its high level (24 h).

To further assess the predictive performance of the reduced model, an independent validation experiment was performed in triplicate under conditions not included in the original design matrix (with *x*_1_, *x*_2_, and *x*_3_ set at level 0 and a reaction time of 24 h; experiments #17, 18, and 19 in Table S1). The experimental conversion was 67.5 ± 5.2% (*n* = 3), which is not significantly different from the model prediction (70.6 ± 6.4%), indicated in the contour plot by the intersection of the two dotted lines. The overlap between the experimental and predicted confidence intervals confirmed the reliability of the model. Additional response surface plots representing other variable combinations were also examined and are collected in Figure S13. These plots display planar surfaces, visually confirming the linear nature of the model and the absence of significant interactions across the entire experimental domain. Based on the model, to maximize monoglyceride production, a low amount of solvent is desirable, while higher amounts of glycerol and enzyme should be used. The reaction should be allowed to proceed for 24 h to ensure optimal yield.

### Novozym435 Stability and Recyclability

The immobilized biocatalyst showed very good stability in TAA. After 48 h of incubation, the enzyme retained 96% of the initial activity, suggesting the possibility to recycle it for further reaction cycles (Figure 3A).

**Figure 3:**
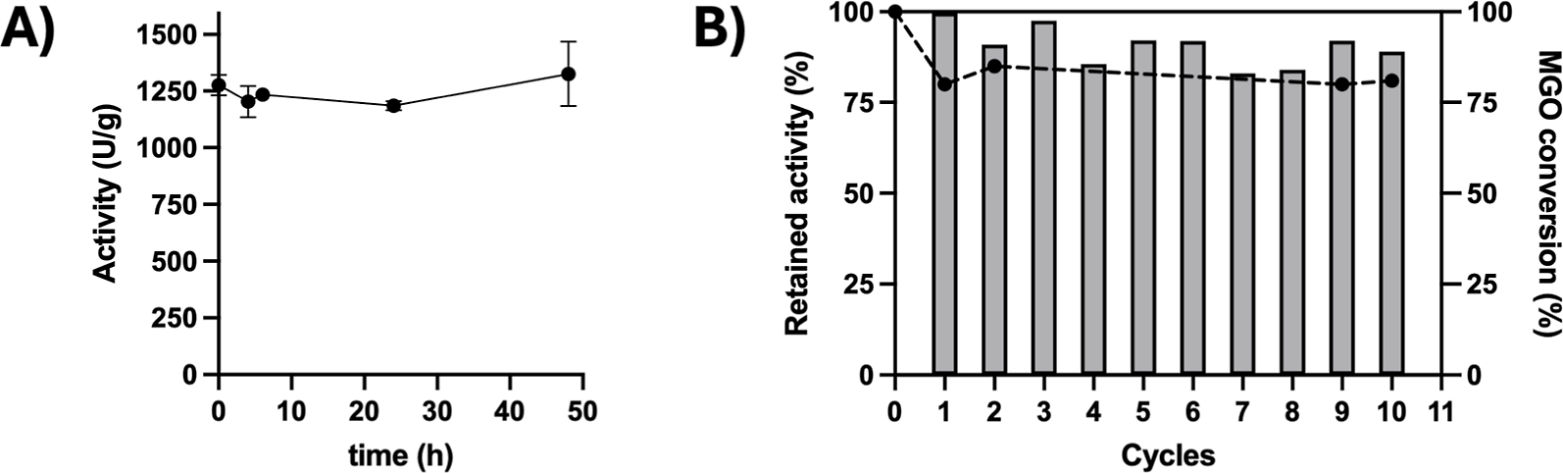
Novozym435 stability in TAA at 50 °C, 400 rpm (A), and recyclability in the optimized reaction conditions (B).

Recycling of immobilized Novozym435 was performed by evaluating the conversion of WCO to MGO (Figure 3B) under the best reaction conditions identified in the DoE study: WCO/solvent ratio 1/1, WCO/glycerol ratio 1/6, 50 °C, 24 h. After each reaction cycle (24 h), a sample of the reaction mixture was taken out to quantify the conversion (HPLC-ELSD), while the biocatalyst was filtered under reduced pressure, washed with TAA, and reused for the following reaction runs. As shown in Figure 3B, the immobilized Novozym435 was successfully reused for 10 cycles with excellent and reproducible conversion (>85%). The activity of the biocatalyst after different cycles was also tested during the recycling study showing also in this case excellent retention of catalytic activity after 10 reaction cycles.

### Preparative Synthesis of Monoacylglycerols from WCO

In order to demonstrate the feasibility of the biocatalytic transformation of WCO and to obtain the products in good amounts to study their interfacial properties, the reaction was scaled up by a factor of 10. The process was carried out under the optimal conditions identified through the DoE study: WCO (5 g), a WCO/solvent w/w ratio of 1/1, a WCO/glycerol molar ratio of 1/6, and an enzyme loading of 935 U (600 mg). After purification by flash chromatography, the monoacylglycerol mixture was obtained in 67% isolated yield. As expected, the fatty acid profile determined by GC–MS closely matched that of the original WCO (Figure 1).

The results obtained indicate that enzymatic glycerolysis of WCO can produce monoacylglycerol-rich mixtures with functional interfacial properties. From a process perspective, some considerations should nevertheless be addressed in view of scale-up and industrial implementation. WCO represents an intrinsically variable feedstock, and its composition may influence the process reproducibility across different batches. In addition, while the use of TAA enhances substrate miscibility and reaction efficiency, solvent recovery and integration will play a role in determining overall cost-effectiveness. Importantly, the comparable emulsifying performance observed for nonpurified and purified MAG fractions suggests that downstream purification steps may be simplified or avoided, partially offsetting process-related costs.

### NIR Analysis

NIR spectroscopy, combined with PCA, was employed to characterize the chemical composition of the enzymatic reaction products and to evaluate the effectiveness of the purification process. The analysis included the nonpurified MAG mixture (the crude product obtained directly after the enzymatic reaction, containing a mixture of MAG and residual glycerol) and the corresponding purified MAG fraction. A selection of commercial reference compounds, including Geleol, monoolein (>40% purity), various FFA, was also analyzed for comparative purposes. It is worth mentioning that the standard reference compound Geleol is a mixture of mono- and diacylglycerols of SA and PA (E471). The HPLC-ELSD analysis confirmed the presence of two different monoacylglycerols, monoglyceryl palmitate (MGP) and monoglyceryl stearate (MGS), but also different diacylglycerol species and traces of triacylglycerol species (Figure S14). Monoolein commercial sample has a declared purity degree >40%. HPLC-ELSD analysis confirmed that the major component of the mixture is monoglyceryl oleate (MGO) with traces of diacylglycerol species (Figure S15). For these reasons we decided to study also the nonpurified MAG mixture. All samples were dispersed in semisolid vaseline prior to NIR analysis and selected as an inert hydrocarbon matrix lacking oxygen-containing functional groups, thus minimizing spectral interference in the NIR region associated with monoacylglycerols. This approach allowed for the identification of diagnostic spectral features and enabled the differentiation of samples based on their compositional profiles. As shown in Figure 4A the NIR absorption band observed around 930 nm is attributed to CH_2_ bonds, indicative of lipid presence.^28^ Both in purified and nonpurified MAG samples this fingerprint signal is detectable, suggesting the presence of lipid tails. The spectral regions near 1400 and 1450 nm correspond to the first overtone of OH stretching vibrations, which is directly associated with the water- or hydroxyl-containing compounds. Absorption bands near 1210 nm are related to the second overtone of C–H vibrations and are characteristic of fatty acid structure.^29^ In the case of glycerol, strong absorption in the 1400–1450 nm region is observed, corresponding to the first overtone of O–H stretching.^30^ Notably, these bands exhibit significantly higher intensity in glycerol than in apolar matrices, such as blank vaseline, where they are almost negligible. The principal component loading plot (Figure 4B) highlights the spectral region between 1400 and 1450 nm as the major contributing variable to the first principal component PC-1. This region, associated with the first overtone of the O–H stretching, reflects the presence of glycerol and other hydroxyl-containing compounds, which contribute negatively to this component. In contrast, a positive contribution is observed around 1200 nm, corresponding to the second overtone of C–H stretching vibrations, typically associated with fatty acid moieties. Variations along the second principal component correspond to spectral changes in the 1600–1650 nm region, at the edge of the analyzed spectral range, suggesting that further investigation is needed to fully elucidate the sample behavior in this region (Figure 4C).

**Figure 4:**
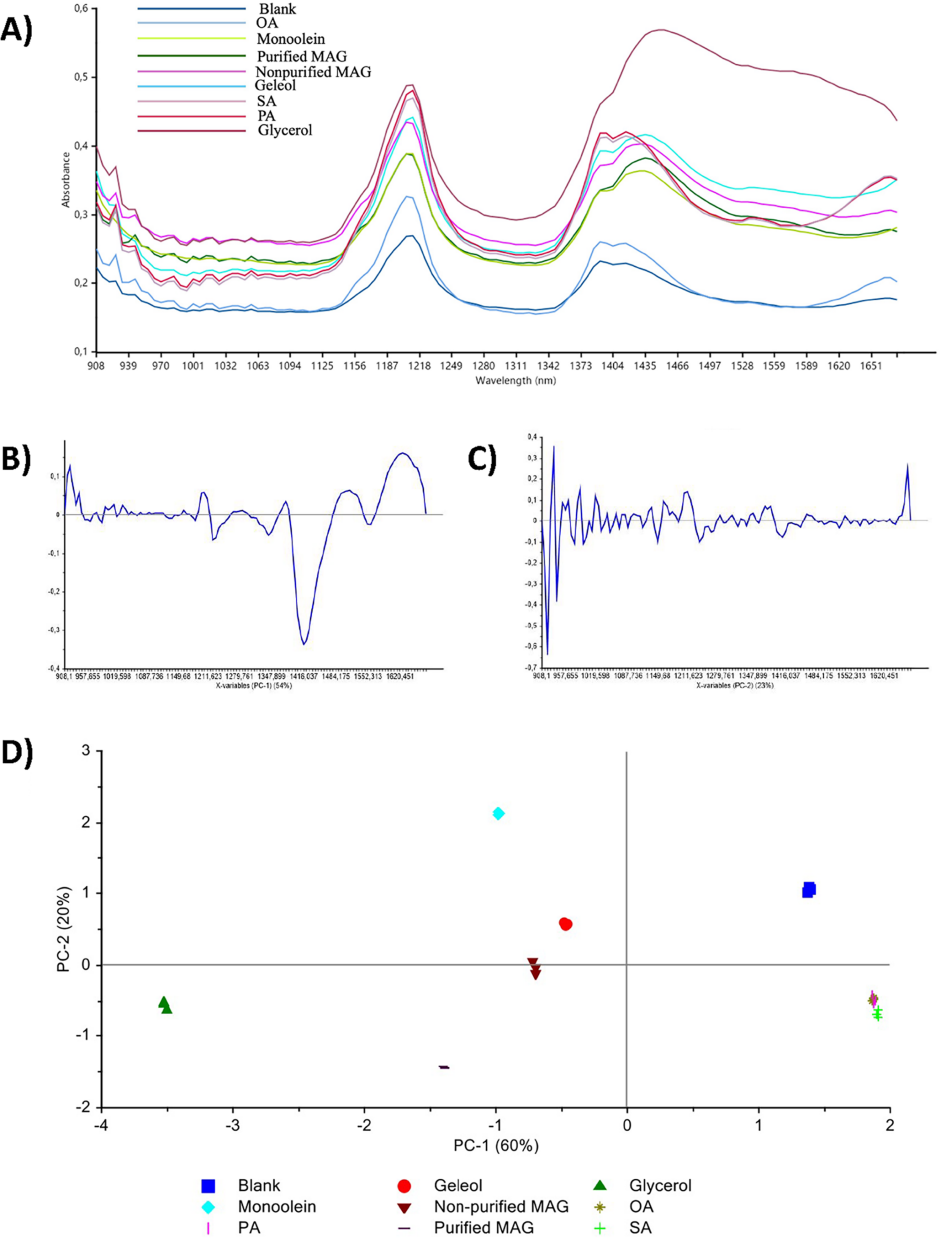
(A) NIR absorbance profiles of the purified monoglyceride (MAG) sample, the nonpurified MAG mixture (crude reaction mixture), and a series of commercial reference compounds: Geleol, monoolein, SA, OA, PA, and glycerol; (B) loading plots of the first (PC-1, explaining 60% of the variance) and (C) second (PC-2, explaining 20% of the variance) principal components obtained from PCA of the NIR spectral data; (D) score plot of PCA analysis illustrating the distribution and clustering of samples based on their NIR spectral data.

The score of PCA analysis (Figure 4D) allows to determine in a qualitative way the main structural differences based on their position. The most polar component glycerol, in the left side, shows the most negative value along PC-1 and the FA are located at the extreme positive side of PC-1. The nonpurified MAG mixture cluster centrally, showing notable structural similarity to the Geleol standard, according to its structure, which is a mixture of MAG and DAG containing as lipid tails both PA and SA. The purified MAG sample shifts to the left in the score plot, indicating compositional differences. Overall, the NIR spectral analysis combined with PCA effectively discriminated samples based on their compositional differences, confirming the successful purification of MAG from the crude reaction mixture. This approach supports the development and monitoring of enzymatic processes for lipid modification and purification.

### Interfacial Tension Measurements

The interfacial analysis was carried out using a mixture of water/sunflower oil (1/1 w/w) with different percentages of surfactants dissolved in the oil phase. The interfacial tension of the blank water/sunflower oil system was 25.03 dyn/cm, consistent with previously reported values.^31^ As shown in Figure 5A, the addition of 0.05 wt % of each surfactant led to a reduction in interfacial tension from 25.0 to 20.4 dyn/cm for the purified MAG sample (corresponding to a 18.6% reduction) and to 21.6 dyn/cm for nonpurified MAG mixture (corresponding to a 13.7% reduction). An interfacial tension decrease is observed in all three cases, with a more significant reduction for purified MAG, in line with the behavior of the commercial Geleol standard (19.3 dyn/cm). The reduction of interfacial tension at 0.1 wt % purified MAG is similar to the one obtained in the presence of the Geleol standard (27% and 28% interfacial tension reduction) while for the nonpurified MAG mixture a lower reduction of interfacial tension is observed (18.7%).

**Figure 5:**
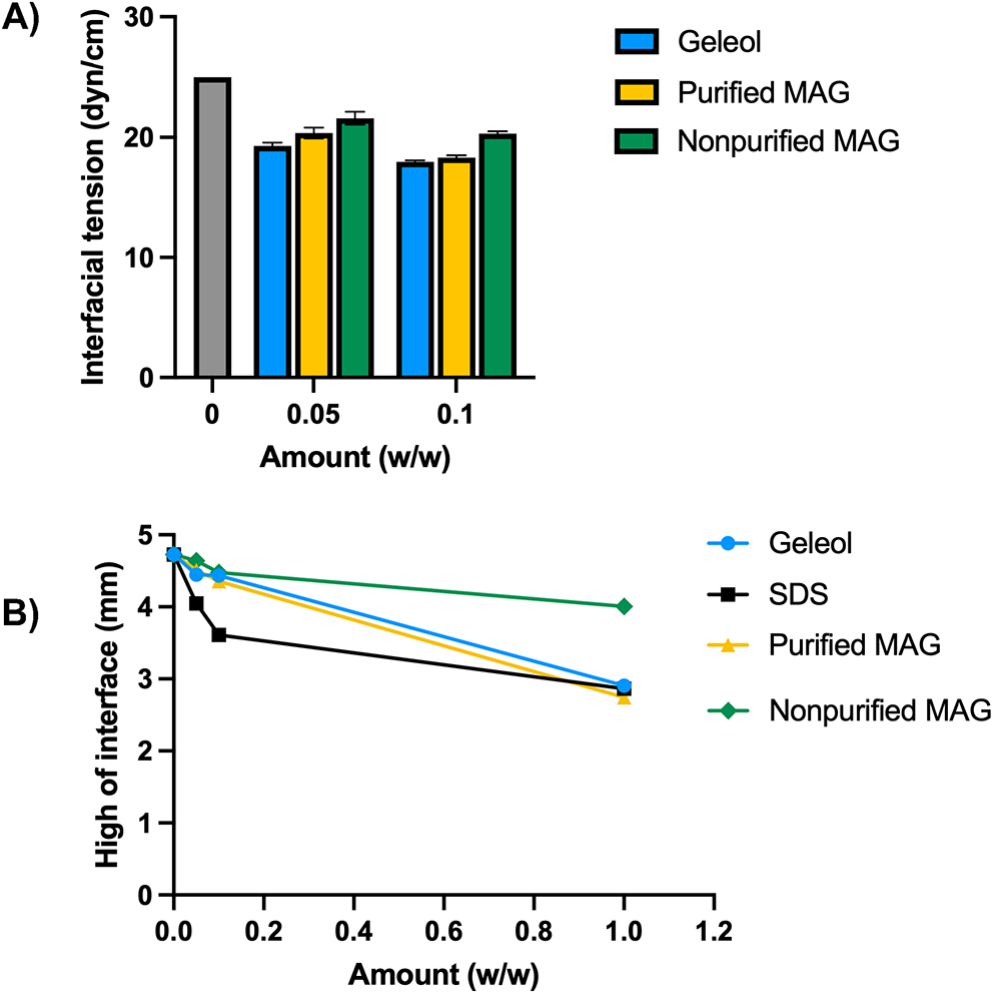
Interfacial properties: (A) Interfacial tension (dyn/cm) of Geleol, purified MAG, and nonpurified MAG samples measured using the Du Noüy ring method and (B) height variation of the interface in different samples assessed by Multiple Light Scattering analysis.

In order to corroborate these promising surface properties, the physical change in the oil/water interface was assessed by multiple light scattering (SMLS) analysis. Detailed oil/water interface variation measurements at different surfactant percentages are shown in Figures S16–S19. As depicted in Figure 5B, the purified MAG decreases the interfacial height from 4.7 to 2.7 mm at 1 wt % allowing to obtain up to 42% decrease compared to the nonpurified sample (just 15.25% interfacial heigh reduction). The ability of purified MAG sample to reduce the interfacial high (42%) is in line with that of reference surfactants with strong interfacial activity: Geleol showed a 38.51% reduction while SDS showed a 39.42% reduction (at 1 wt %). These results confirm that the purified MAG sample possesses significant surface-active properties, which are notably reduced in the crude mixture due to the presence of other less active components (i.e., glycerol).

### Stability Analysis

Finally, O/W emulsions were prepared by homogenization of lipophilic and hydrophilic phases added with different surfactants (purified MAG, nonpurified MAG, Geleol) and their stability was evaluated by multiple light scattering within 3 days of incubation (every 30 min) at 40 °C.

As shown in Figure 6 the backscattering profiles of emulsions formulated with Geleol as a standard and the purified MAG sample showed highly similar behavior over the 3-day analysis period at 40 °C. In both cases, the variation in backscattering remained below 5%, indicating excellent physical stability. The signal peak observed on the right side of the graph corresponds to the air/emulsion interface and is not considered relevant for stability evaluation. In contrast, the formulation containing the nonpurified MAG mixture exhibited a markedly different backscattering profile (Figure 6C). A pronounced peak near the bottom of the vial, visible on the left side of the profile, indicates the onset of the destabilization phenomena. Interestingly, this destabilization signal appears to diminish over time, suggesting a partial reorganization or limited recovery of the system. This behavior is likely due to the heterogeneous composition of the nonpurified MAG mixture, reducing the overall emulsifying efficiency compared to the purified MAG sample.

**Figure 6:**
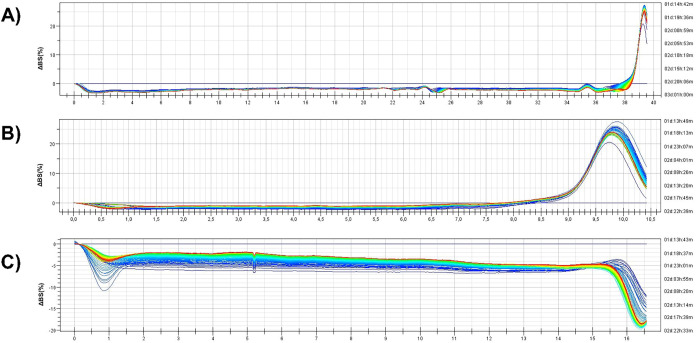
Δ Backscattering profile over 3 days analyses at 40 °C of the formulation containing (A) Geleol (B) purified MAG sample and (C) the nonpurified MAG mixture.

This work demonstrates the feasibility of enzymatically converting WCO into high-value MAG using a sustainable and efficient biocatalytic process. Through a systematic DoE-based optimization, experimental conditions were identified that maximize MAG yield (67%), employing TAA as a green cosolvent. The use of TAA significantly improved substrate miscibility, enhancing the reaction efficiency and minimizing side-product formation. The resulting MAG preserved the fatty acid composition of the starting WCO, predominantly oleic and linoleic acids, and showed excellent interfacial activity, comparable to commercial emulsifiers used in food applications. These properties suggest their potential as a more sustainable alternative to E471, especially considering the lower content of saturated fatty acids. While E471 contains stearic and palmitic acids derived from less sustainable sources, the biobased MAG produced in this work may offer both functional and nutritional benefits. Overall, this process demonstrates a successful integration of waste valorization, biocatalysis, and green chemistry principles. It represents a promising strategy for producing functional food emulsifiers from an otherwise problematic waste stream, in alignment with circular economy models and sustainable food ingredient manufacturing.

## Supplementary Material



## Notes

Calibration curve of purified standard monoglyceryl oleate (MGO), comparative HPLC analysis of 1 mg/mL solutions of monoglyceryl oleate (MGO) and DGO derived from the purification of the commercial MGO, PA, OA, and SA and WCO, comparative TLC: MAG, DAG, OA, WCO, HPLC profile of WCO (1 mg/mL), ATR-FTIR spectrum of waste oil (green) compared with sunflower oils used for method calibration, FTIR first-derivative spectra processed with a Savitzky–Golay filter, screening of the amount of enzyme, preliminary solvent screening, preliminary WCO/glycerol ratio screening, preliminary WCO/solvent ratio screening, full factorial design (2^4^ = 16 experiments) reporting the combinations of the four studied parameters and the response variable for each point, ANOVA results and regression coefficients of the full factorial model, screening of significant variables: Pareto chart of the standardized effects for the full factorial model, ANOVA results and regression coefficients of the reduced linear model including only significant main effects, comparative parity plot of experimental versus predicted conversion values derived from the final reduced model, 3D Response surface plots for the reduced model illustrating the interaction between factor pairs while holding the other variables constant at their center point, HPLC chromatogram of glyceryl stearate (Geleol) (1 mg/mL), HPLC chromatogram of monoolein (TCI) (>40% purity) (1 mg/mL), change in the transmission profile of the oil/water interface height with different percentages (w/w) of purified MAG, change in the transmission profile of the oil/water interface height with different percentages (w/w) of nonpurified MAG, change in the transmission profile of the oil/water interface height with different percentages (w/w) of Geleol, and change in the transmission profile of the oil/water interface height with different percentages (w/w) of SDS (PDF)
