# Supplementary material for: Sustainable Synthesis of Food-Grade Emulsifiers from Waste Cooking Oil via Enzymatic Glycerolysis in a Green Solvent System
Source: J Agric Food Chem. 2026 Feb 7;74(6):5527–38. doi: 10.1021/acs.jafc.5c14323 (PMC12921849; doi:10.1021/acs.jafc.5c14323)
Supplement: Supplementary file 1 [file jf5c14323_si_001.pdf]

SUPPORTING INFORMATION

Sustainable Synthesis of Food-Grade Emulsifiers from Waste Cooking Oil via Enzymatic Glycerolysis in a Green Solvent System

Stefano Genualdo,[a,#] Marina Simona Robescu,[a,#] Sara Tengattini,[a] Vitiana Cerone,[a,b] Dhanalakshmi Vadivel,[c] Paola Perugini,[a,b] Daniele Dondi,[c] Teodora Bavaro[a,]*

[a]Department of Drug Sciences, University of Pavia, Viale Taramelli 12, 27100 Pavia, Italy
[b]Etichub s.r.l., Academic Spin-Off, University of Pavia, Viale Taramelli 12, 27100 Pavia, Italy
[c]Department of Chemistry, University of Pavia, Viale Taramelli 12, 27100 Pavia, Italy
*Correspondence: teodora.bavaro@unipv.it;
[#]These authors contributed equally to this work and share first authorship.

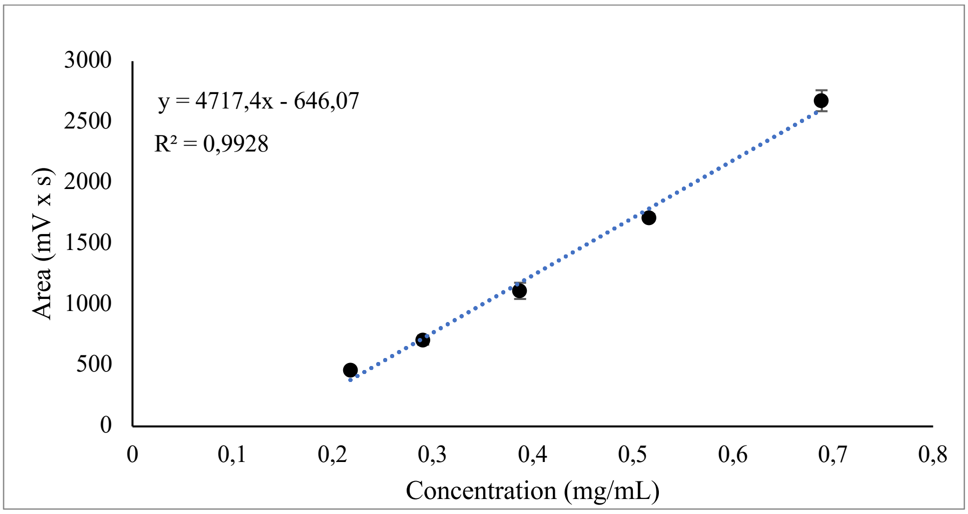

Figure S1. Calibration curve of purified standard monoglyceryl oleate (MGO).

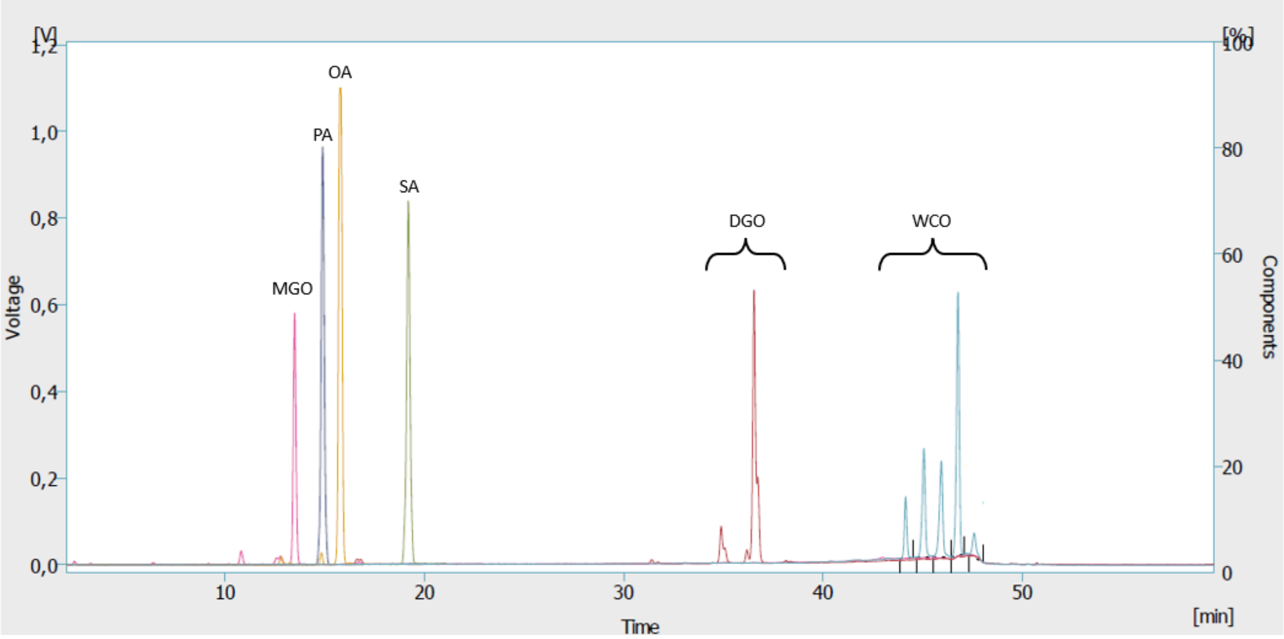

Figure S2. Comparative HPLC analysis of 1 mg/mL solutions of monoglyceryl oleate (MGO) and diglyceryl oleate (DGO) derived from the purification of the commercial MGO, the three FFA (palmitic acid (PA), oleic acid (OA) and stearic acid (SA)) and WCO.

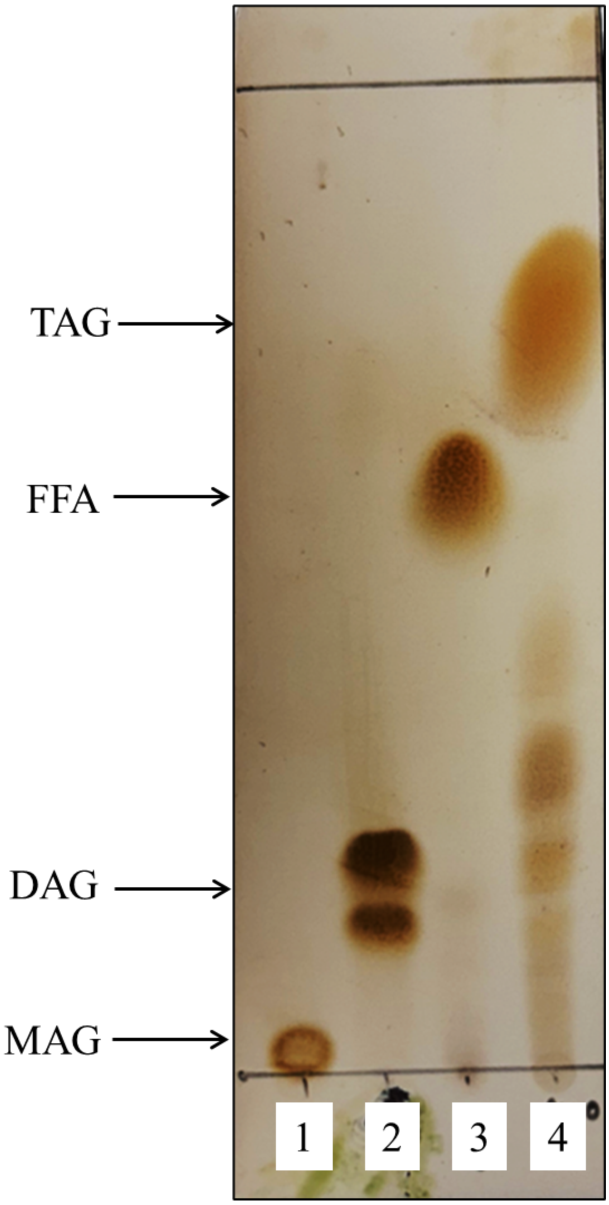

Figure S3. Comparative TLC: 1) MAG; 2) DAG; 3) Oleic Acid; 4) WCO. 3 μL of each compound were spotted for TLC analysis (*n*-hexane/diethyl ether 8:2 with 0.02% formic acid).

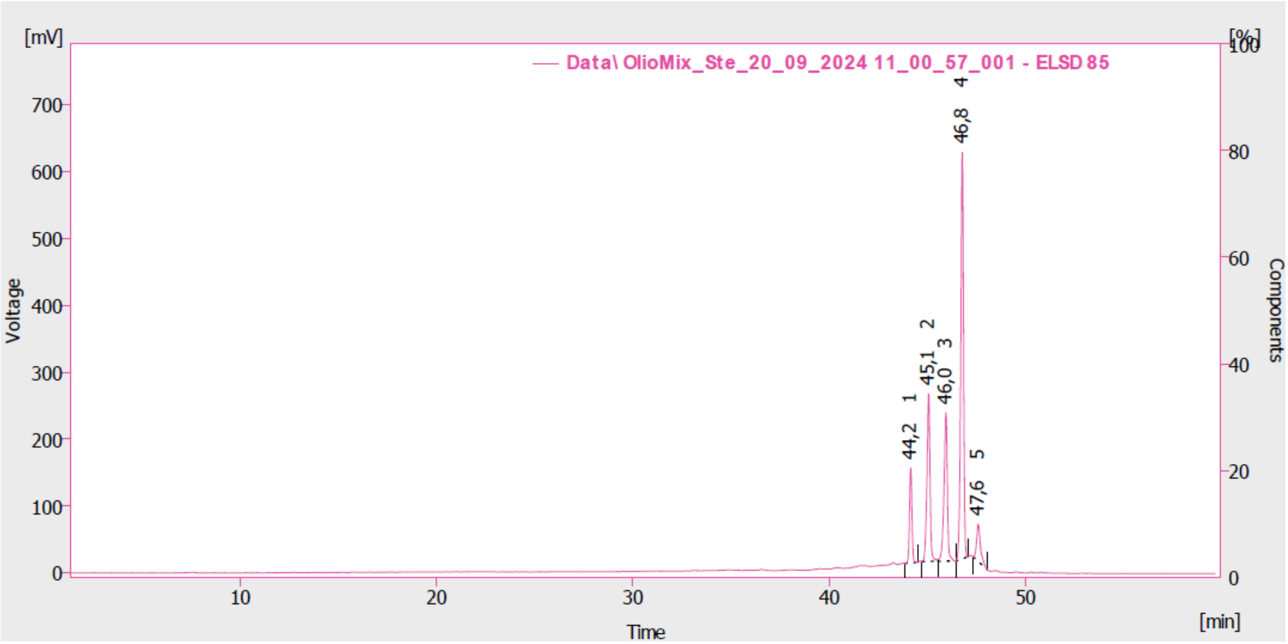

Figure S4. HPLC profile of WCO (1 mg/mL).

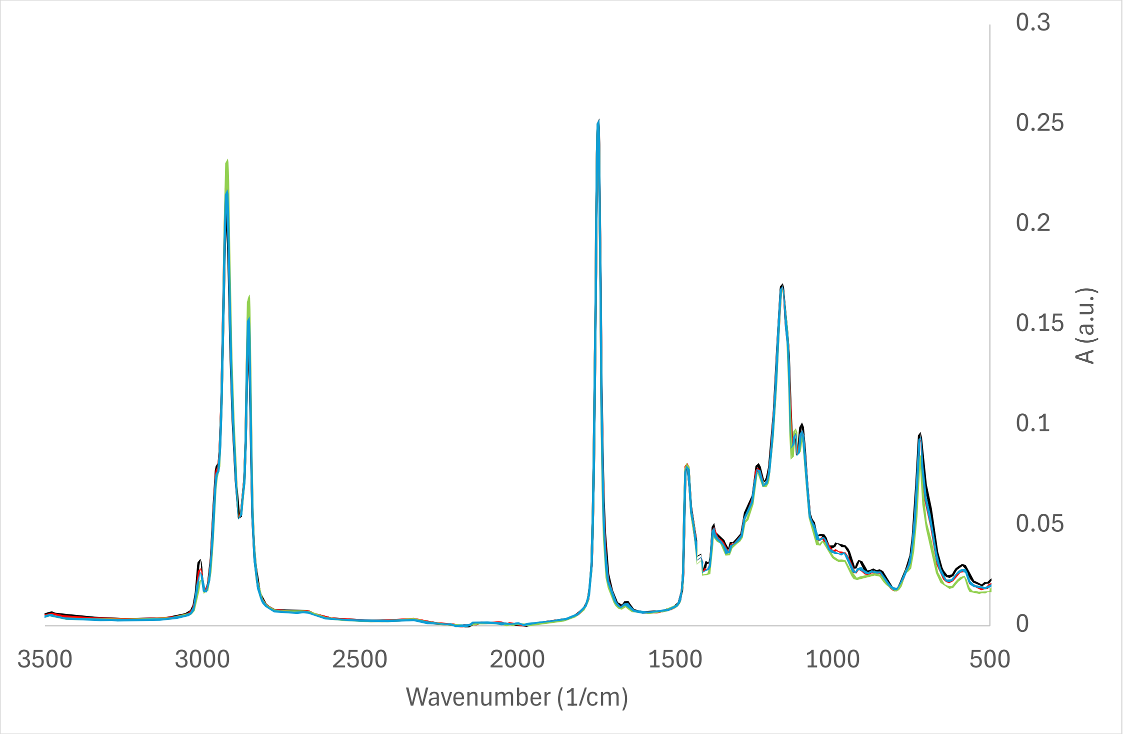

Figure S5. ATR–FTIR spectrum of waste oil (green) compared with sunflower oils used for method calibration.

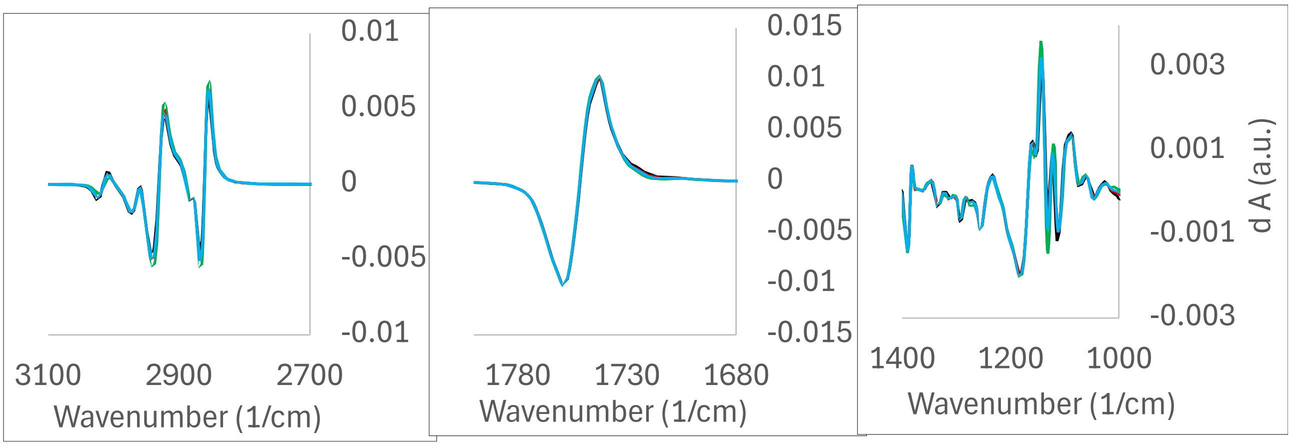

Figure S6. FTIR first-derivative spectra processed with a Savitzky–Golay filter. Green: waste cooking oil used in this work compared with sunflower oils used for method calibration.

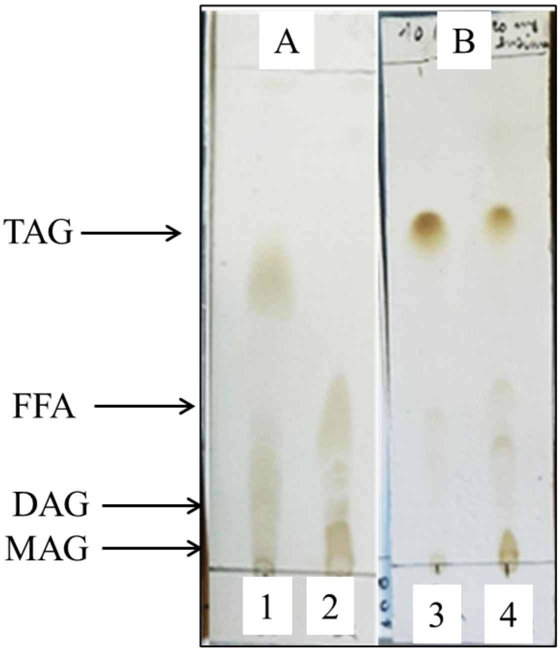

Figure S7. Screening of the amount of enzyme. A) WCO (1 g) reaction with 200 mg (312 U) of enzyme monitored at t0 (1) and after 24 h (2); B) WCO (0.5 g) reaction with 20 mg (31 U) of enzyme monitored at t0 (1) and after 24 h (2). Reactions were incubated for 24 h at 50 °C and 3 µL of each reaction were spotted for TLC analysis (*n*-hexane/diethyl ether 8:2 with 0.02% formic acid).

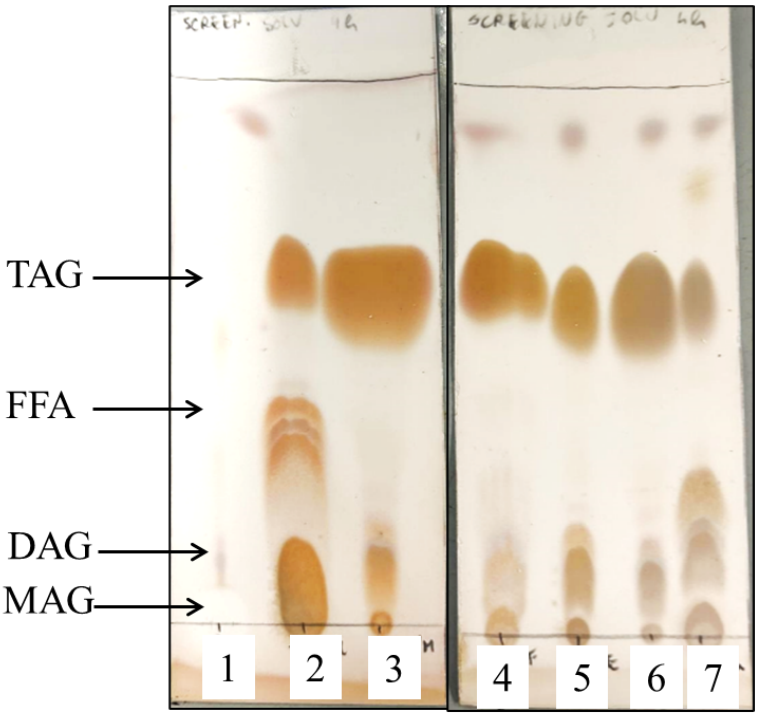

Figure S8. Preliminary solvent screening. The solvents screened are: glycerol formal (1), *tert*-amyl alcohol (2), *p*-cymene (3), 2-methyl tetrahydrofuran (4), methyl *tert*-butyl ether (5), isopropanol (6) and *tert*-butanol (7). Reactions were incubated for 4 h at 50 °C and 3 µL of each reaction were spotted for TLC analysis (*n*-hexane/diethyl ether 8:2 with 0.02% formic acid).

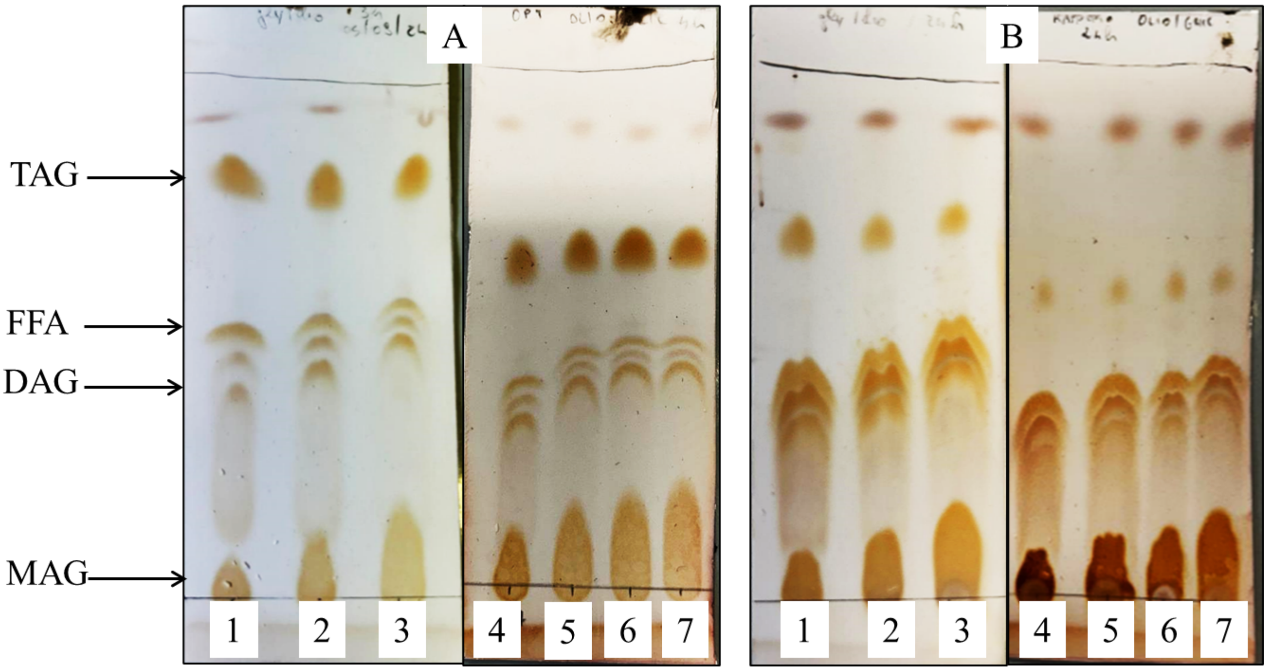

Figure S9 Preliminary WCO/glycerol ratio screening. A) 2 h monitoring of WCO/glycerol 1:2 (1), WCO/glycerol 1:3 (2), WCO/glycerol 1:5 (3), WCO/glycerol 1:7 (4), WCO/glycerol 1:8 (5), WCO/glycerol 1:9 (6), WCO/glycerol 1:10 (7). B) 24 h monitoring of WCO/glycerol 1:2 (1), WCO/glycerol 1:3 (2), WCO/glycerol 1:5 (3), WCO/glycerol 1:7 (4), WCO/glycerol 1:8 (5), WCO/glycerol 1:9 (6), WCO/glycerol 1:10 (7). Reactions were monitored after 2 h and 24 h incubation at 50 °C and 3 μL of each reaction were spotted for TLC analysis (*n*-hexane/diethyl ether 8:2 with 0.02% formic acid).

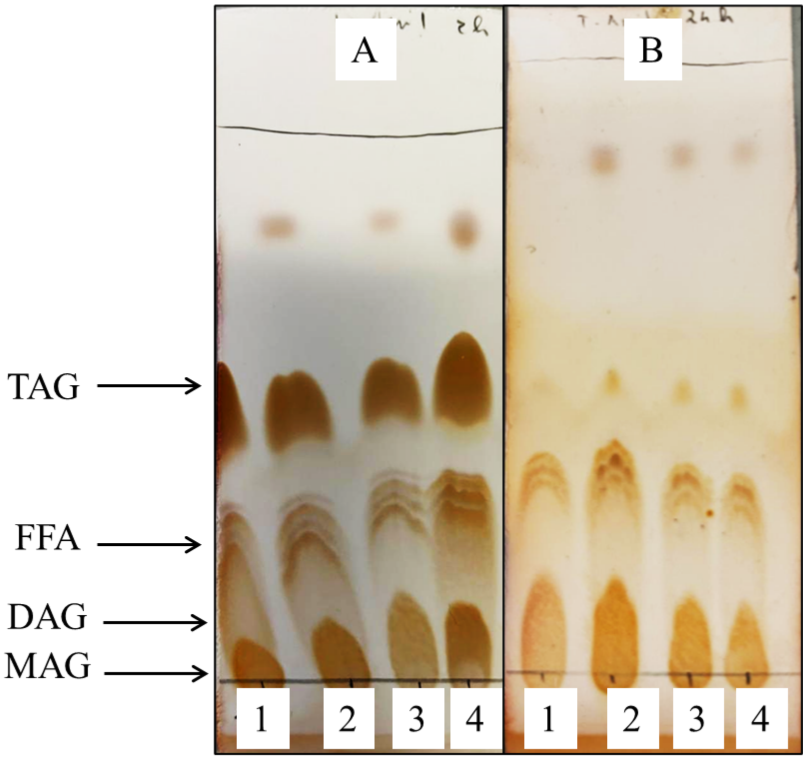

Figure S10. Preliminary WCO/solvent ratio screening. A) 2 h monitoring of WCO/solvent 1:5 (1), 50% less solvent (2), 75% less solvent (3), 85 % less solvent (4); B) 24 h monitoring of WCO/solvent 1:5 (1), 50% less solvent (2), 75% less solvent (3), 85 % less solvent (4). Reactions were monitored after 2 h and 24 h incubation at 50 °C and 3 μL of each reaction were spotted for TLC analysis (*n*-hexane/diethyl ether 8:2 with 0.02% formic acid).

Table S1. Full factorial design ($2^4 = 16$ experiments) reporting the combinations of the four studied parameters and the response variable for each point. Three additional reaction were performed at the central point (CD) to evaluate model's predictivity.

Experiment	WCO/solvent (w/w)	WCO/glycerol (mol/mol)	Enzyme/WCO (mg/g)	Reaction time (h)	Conversion* (%)
1	1/5	1/6	120	6	66.0
2	1/1	1/6	120	6	79.5
3	1/5	1/2	120	6	41.3
4	1/1	1/2	120	6	51.8
5	1/5	1/6	20	6	30.0
6	1/1	1/6	20	6	51.1
7	1/5	1/2	20	6	14.2
8	1/1	1/2	20	6	38.9
9	1/5	1/6	120	24	72.0
10	1/1	1/6	120	24	>99
11	1/5	1/2	120	24	54.4
12	1/1	1/2	120	24	>99
13	1/5	1/6	20	24	68.9
14	1/1	1/6	20	24	73.1
15	1/5	1/2	20	24	41.3
16	1/1	1/2	20	24	57.3
17 (CP)	1/3	1/4	70	24	73.5
18 (CP)	1/3	1/4	70	24	64.4
19 (CP)	1/3	1/4	70	24	64.7

*Conversion was calculated by taking into consideration the 45% content of the WCO in oleic acid fatty acid tails.

Table S2. Analysis of variance (ANOVA) results and regression coefficients of the full factorial model.

Source	Degree of freedom	Coefficient	Std. dev.	Conf. Int.	F-Value	p-Value
Model	10					
WCO/solvent (x1)	1	-10.10	2.63	6.76	14.73	**0.0121**
WCO/glycerol (x2)	1	8.84	2.63	6.76	11.28	**0.0201**
Enzyme/WCO (x3)	1	11.76	2.63	6.76	19.99	**0.0066**
Reaction time (x4)	1	12.01	2.63	6.76	20.84	**0.0060**
x1x2	1	1.88	2.63	6.76	0.51	0.5080
x1x3	1	-1.85	2.63	6.76	0.49	0.5134
x1x4	1	-1.38	2.63	6.76	0.27	0.6236
x2x3	1	-0.09	2.63	6.76	0.01	0.9748
x2x4	1	-1.21	2.63	6.76	0.21	0.6643
x3x4	1	-1.29	2.63	6.76	0.24	0.6453
Error (Residuals)	5					
% Explained Variance (R^2): 79.61						
% CV Explained Variance (Q^2): 34.75						
Root Mean Square Error (RMSE): 10.53						

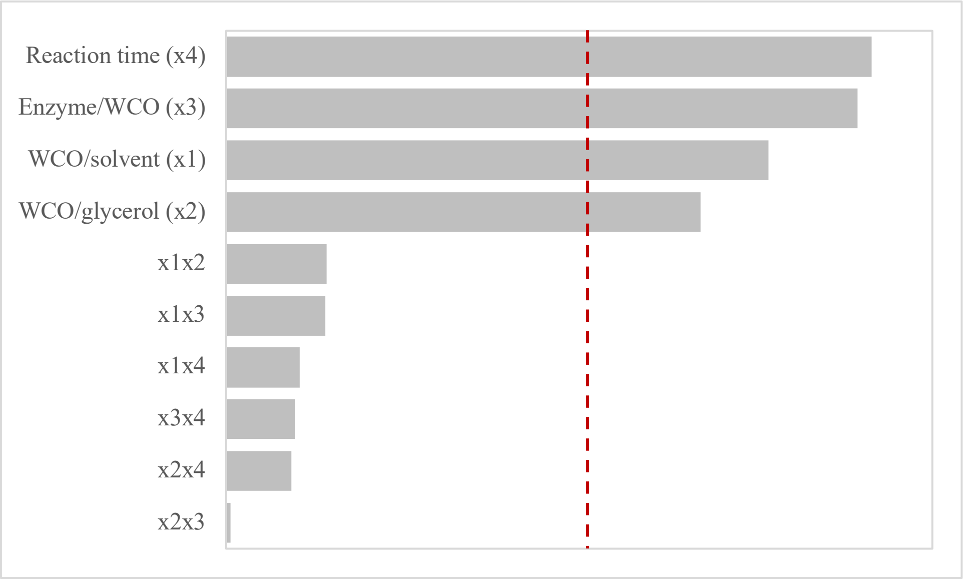

Figure S11. Screening of significant variables: Pareto chart of the standardized effects for the full factorial model. The vertical line represents the critical t-value (t_{limit} = 2.571) at a 95% confidence level (p = 0.05). Only the main effects exceed the significance limit, indicating statistically significance, while all interaction terms are non-significant.

Table S3. ANOVA results and regression coefficients of the reduced linear model including only significant main effects.

Source	Degree of freedom	Coefficient	Std. dev.	Conf. Int.	F-Value	p-Value
Model	4					
WCO/solvent (x1)	1	-10.10	2.06	4.53	24.11	**0.0005**
WCO/glycerol (x2)	1	8.84	2.06	4.53	18.46	**0.0013**
Enzyme/WCO (x3)	1	11.76	2.06	4.53	32.72	**0.0001**
Reaction time (x4)	1	12.01	2.06	4.53	34.11	**0.0001**
Error (Residuals)	11					
% Explained Variance (R^2): 87.53						
% CV Explained Variance (Q^2): 81.86						
Root Mean Square Error (RMSE): 8.23						

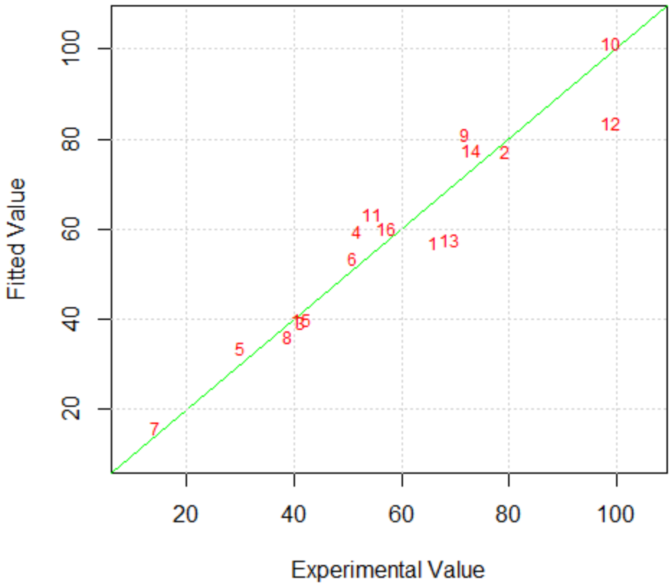

Figure S12. Comparative parity plot of experimental versus predicted conversion values derived from the final reduced model. The solid diagonal line represents the perfect fit. The close distribution of data points around the diagonal confirms the good reliability and accuracy of the regression model.

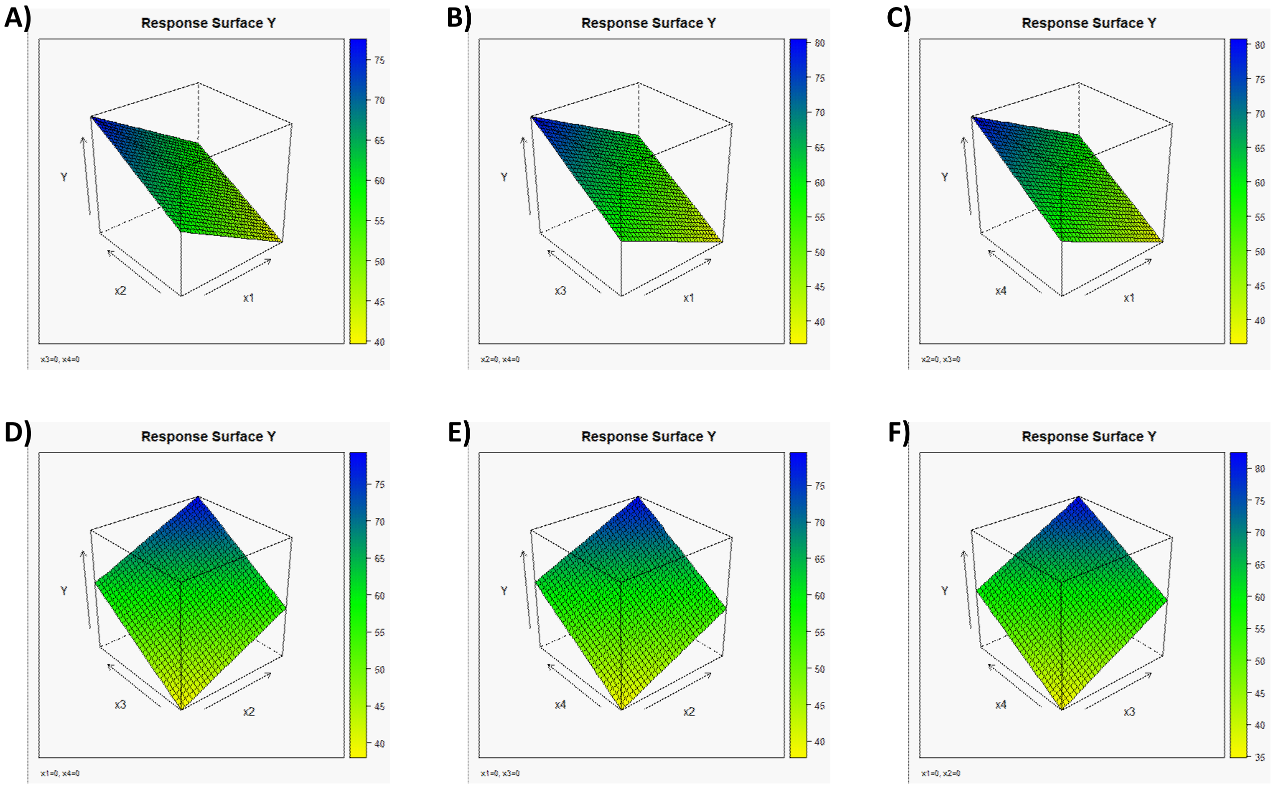

Figure S13. 3D Response surface plots for the reduced model, illustrating the interaction between factor pairs while holding the other variables constant at their centre point: A) x_1x_2; B) x_1x_3; C) x_1x_4; D) x_2x_3; E) x_2x_4; F) x_3x_4. The planar shape of all surfaces visually confirms the linear nature of the model and the absence of significant interactions across the entire experimental domain.

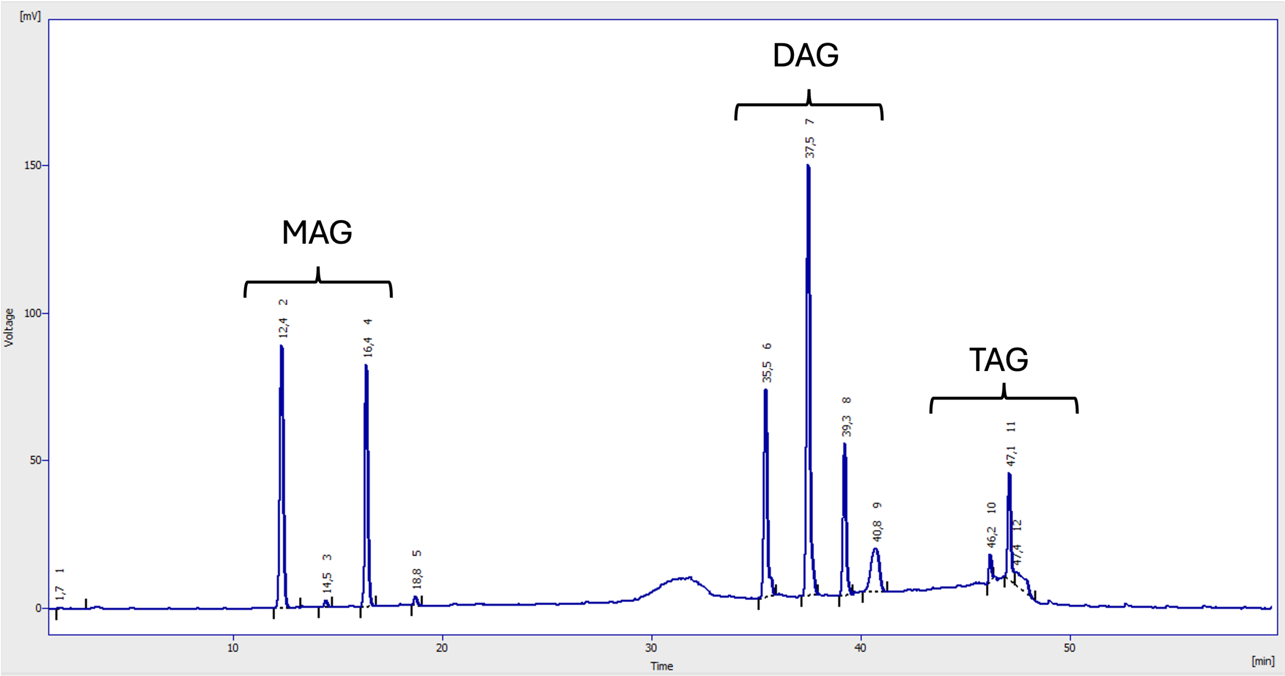

Figure S14. HPLC chromatogram of glyceryl stearate (Geleol) (1 mg/mL).

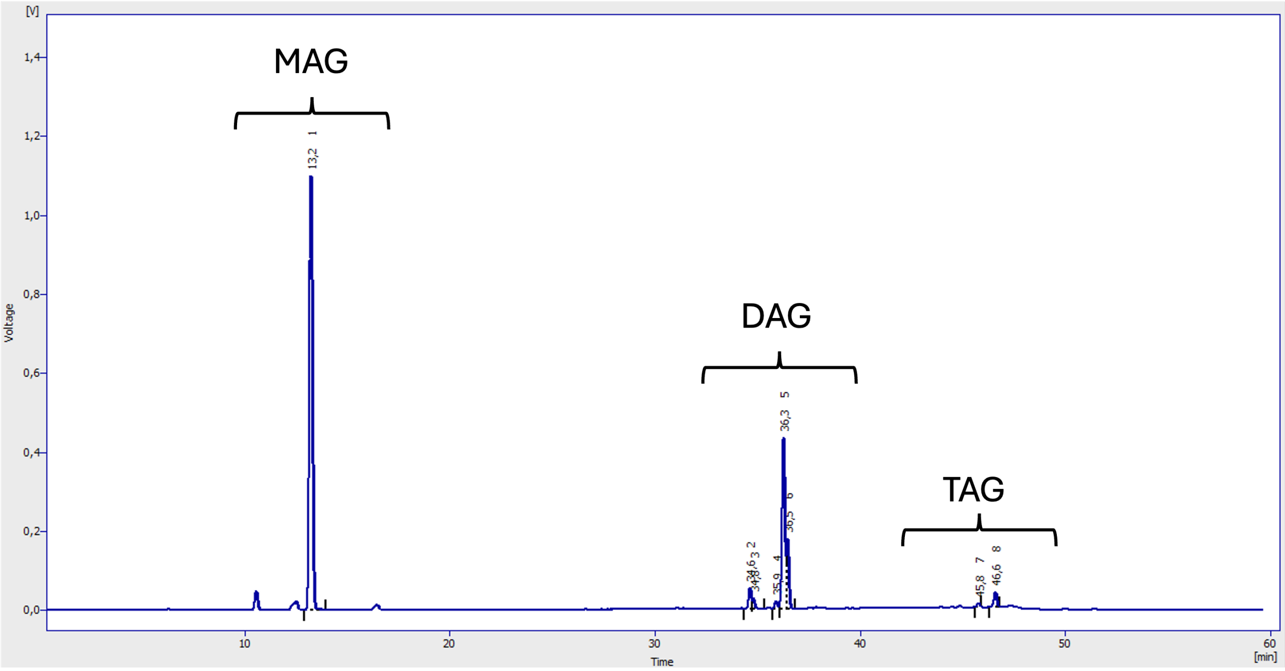

Figure S15. HPLC chromatogram of monoolein (TCI) (>40% purity) (1 mg/mL).

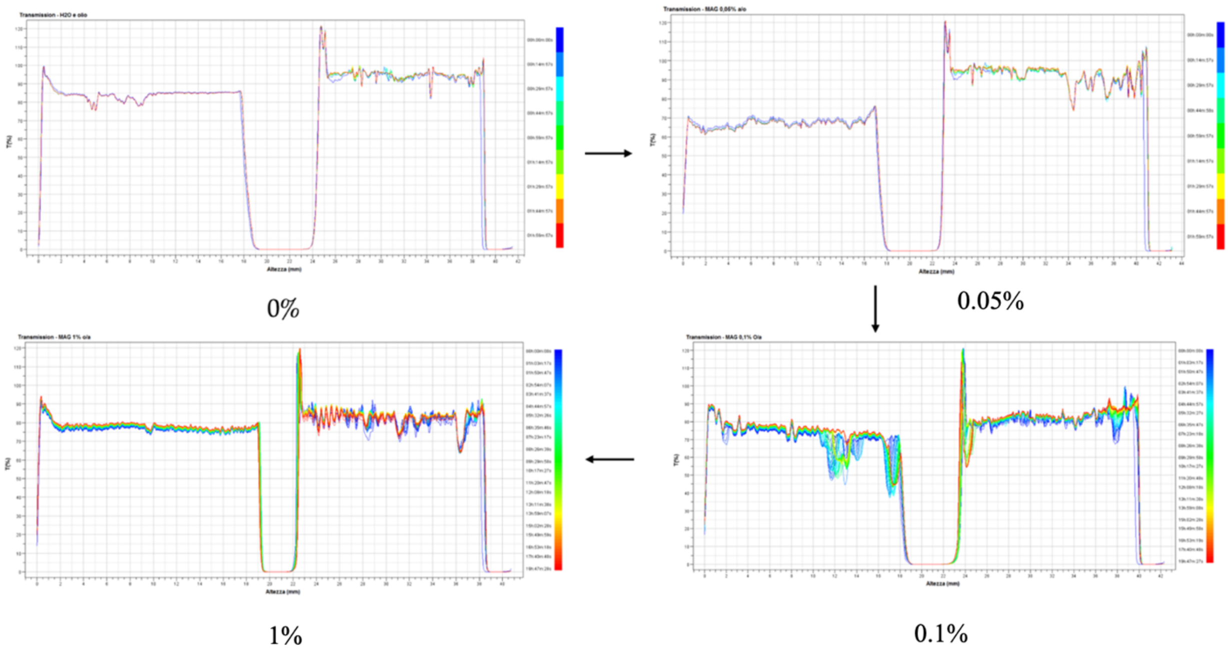

Figure S16. Change in the transmission profile of the oil/water interface height with different percentages (w/w) of purified MAG.

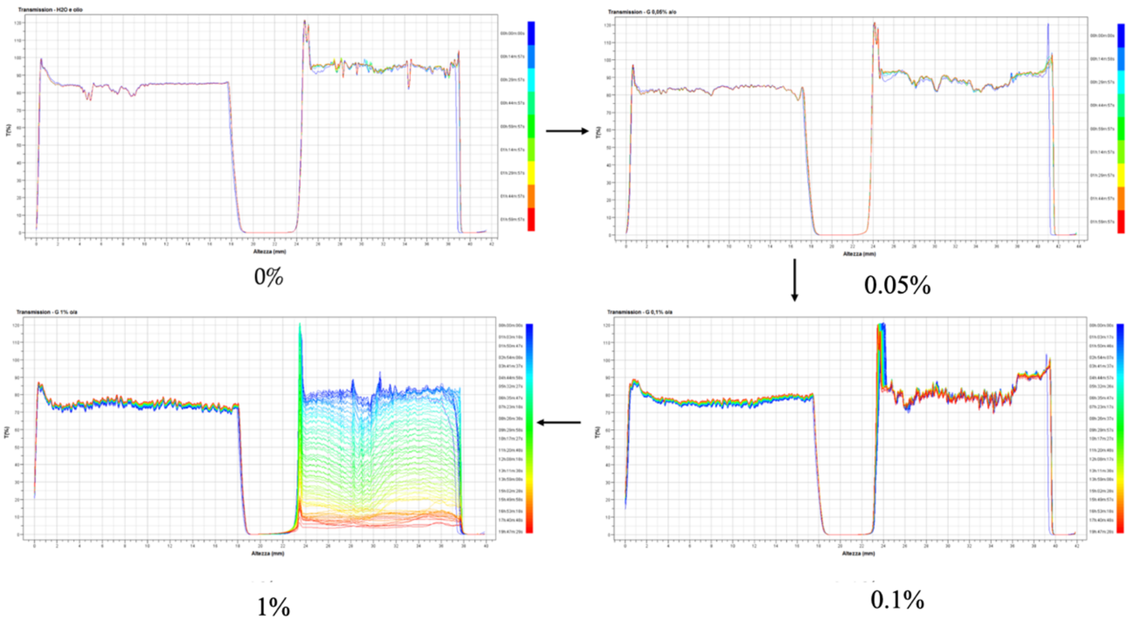

Figure S17. Change in the transmission profile of the oil/water interface height with different percentages (w/w) of nonpurified MAG.

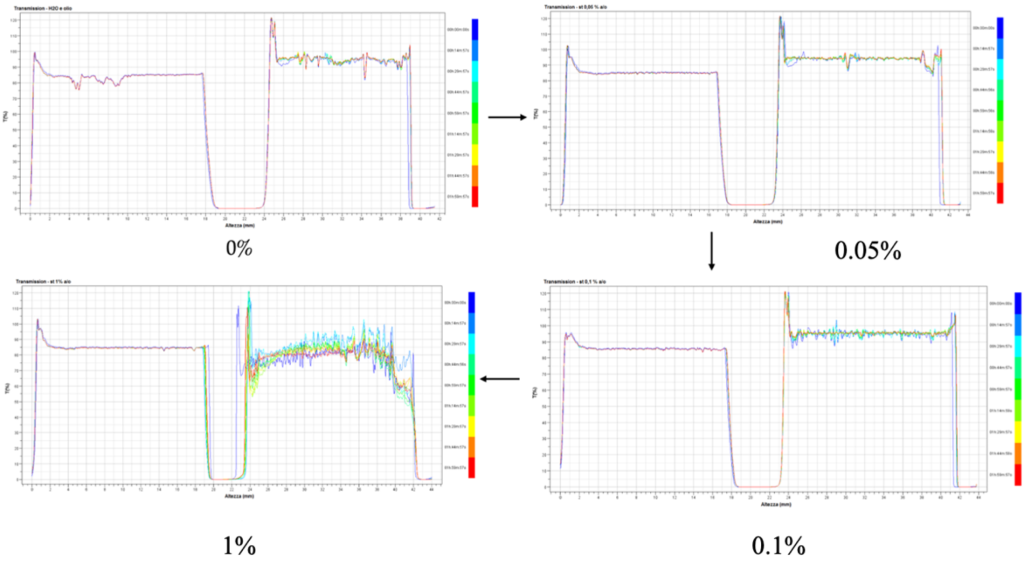

Figure S18. Change in the transmission profile of the oil/water interface height with different percentages (w/w) of Geleol.

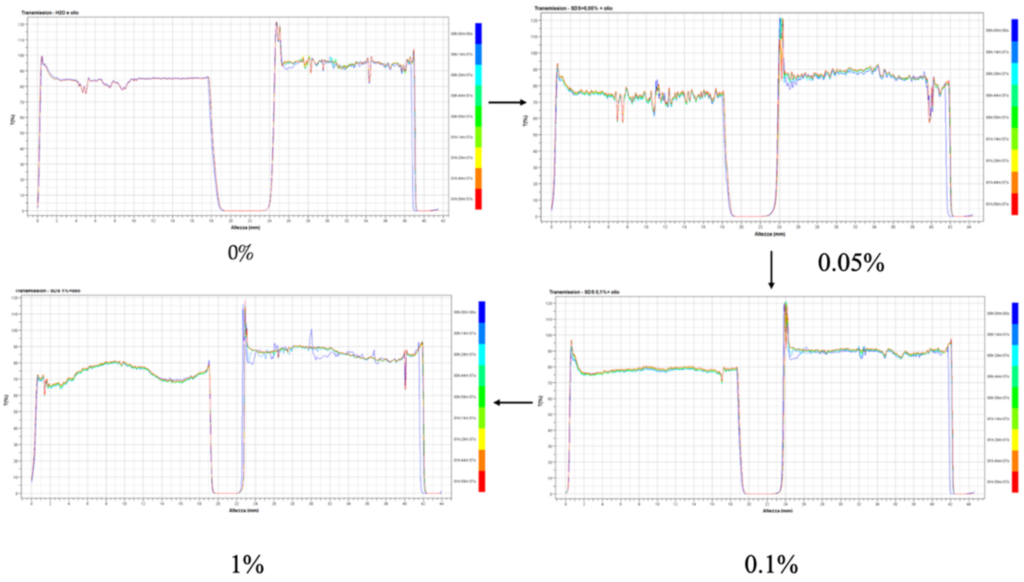

Figure S19. Change in the transmission profile of the oil/water interface height with different percentages (w/w) of sodium dodecyl sulphate (SDS).
